# Exploring sex-specific symptom trajectories in psychosis following metacognitive training (MCT): Evidence from a large-scale retrospective harmonized database

**DOI:** 10.1371/journal.pone.0356632

**Published:** 2026-09-21

**Authors:** Maria Lamarca, Adrien Goncalves, Steffen Moritz, Łukasz Gawęda, Vanessa Acuña, Caroline König, Fabrice Berna, Susana Ochoa

**Affiliations:** 1 Parc Sanitari Sant Joan de Déu, Sant Boi de Llobregat, Barcelona, Spain; 2 Grup MERITT, Fundació Sant Joan de Déu, Institut de Recerca Sant Joan de Déu, Esplugues de Llobregat, Barcelona, Spain; 3 Consorcio de Investigación Biomédica en Red de Salud Mental (CIBERSAM), Instituto de Salud Carlos III, Madrid, Spain; 4 Departament de Psicologia Clínica i de la Salut, Facultat de Psicologia, Universitat Autònoma de Barcelona, Bellaterra, Cerdanyola del Vallès, Barcelona, Spain; 5 University of Strasbourg, Inserm, UMR_S 1329, Strasbourg, France; 6 Department of Psychiatry and Psychotherapy, University Medical Center Hamburg, Hamburg, Germany; 7 Experimental Psychopathology Lab, Institute of Psychology, Polish Academy of Sciences, Warsaw, Poland; 8 Departamento de Psiquiatría, Escuela de Medicina, Facultad de Medicina, Universidad de Valparaíso, Valparaíso, Chile; 9 Soft Computing Research Group at Intelligent Data Science and Artificial Intelligence Research Center, Center Universitat Politècnica de Catalunya, Barcelona, Spain; 10 University Hospital of Strasbourg, Strasbourg, France; University Hospital Marques de Valdecilla, SPAIN

## Abstract

Sex-related differences in the clinical presentation and course of schizophrenia spectrum disorders are increasingly recognized, yet it remains unclear whether biological sex moderates response to psychological interventions. Metacognitive Training for psychosis is an evidence-based psychological intervention targeting cognitive biases associated with psychotic symptoms, but evidence regarding sex-specific treatment effects remain limited. The present study examined whether biological sex influences symptom trajectories following Metacognitive Training in individuals with schizophrenia spectrum disorders. We analyzed harmonized retrospective data from an international consortium (PERMEPSY) including participants who received Metacognitive Training for psychosis in routine clinical or research settings. The sample comprised 573 participants diagnosed with schizophrenia spectrum disorders across different stages of illness. Control group data was not available due to the retrospective nature of the data and a lack of a standardised control group among included datasets. Psychotic symptoms were assessed using the Positive and Negative Syndrome Scale, including positive, negative, general, and total symptom scores. Repeated-measures analyses of variance were conducted to examine the effects of time, sex, and their interaction on symptom change. Across the full sample, Metacognitive Training for psychosis was associated with significant reductions in positive, negative, general, and overall psychotic symptoms. However, no significant moderation of symptom change by biological sex was detected, indicating comparable symptom improvement in males and females across all symptom domains. These findings are consistent with prior evidence supporting MCT as a broadly applicable psychological intervention for psychosis, with comparable symptom changes observed in males and females following treatment. Although sex-related differences in baseline symptom profiles were observed, biological sex did not influence the trajectory of symptom change following treatment. These results support the generalizability of Metacognitive Training and point to the relevance of examining more nuanced sex-related factors in future research.

## Introduction

Schizophrenia spectrum disorders (SSD) is a severe mental health condition characterized by hallucinations, delusions, and disorganized thinking [1]. Biological sex-specific differences in psychosis have been consistently observed across clinical presentation, course of illness, and treatment response [2–4]. Males with schizophrenia tend to experience earlier onset, more negative symptoms, fewer affective symptoms, worse psychosocial functioning, including more social isolation, and higher rates of substance use. Conversely, females often present with more affective symptoms, such as depression and anxiety, resulting in higher rates of schizoaffective diagnoses, and generally demonstrate better premorbid and social functioning [2,5]. However, regarding cognitive and psychosocial functioning, sex differences are complex and heterogeneous: females have been shown to exhibit lower performance across several cognitive domains, including current and premorbid intellectual functioning, working memory, and non-verbal abstract reasoning, while demonstrating preserved or superior abilities in processing speed and verbal learning [6]. These differences are often particularly pronounced during the early stages of the disorder [3]. Consistent with these findings, a recent meta-analysis of first-episode psychosis reported that males exhibited more pronounced negative symptoms, whereas females had higher functioning and more depressive symptoms [4]. Such sex-related differences in clinical presentation may contribute to distinct recovery trajectories in males and females.

Beyond clinical presentation, sex differences also extend to treatment response. With respect to pharmacological interventions, females generally respond better to antipsychotics, requiring lower doses and experiencing fewer relapses, although they are more susceptible to side effects like hyperprolactinemia and weight gain [3,7]. Psychological interventions, including cognitive-behavioral and metacognitive therapies, have likewise been shown to display sex-related differences in effectiveness. For instance, a recent study found that females with first-episode psychosis (FEP) showed higher rates of functional remission and fewer hospitalizations over time compared to males, suggesting potential sex-related variability in response to early psychological treatments [8]. Similarly, some reports suggest females treated in early intervention services for psychosis were more likely to achieve symptom remission and better functioning than males after two years of treatment, responded more favourably to minimal antipsychotic dosages, and showed higher recovery rates during the first three years of early intervention, including psychological support [9,10]. Although premorbid functioning and other risk factors partially account for these differences, they nonetheless suggest the presence of sex-specific outcomes in psychological care [11]. Moreover, sex differences in predictors of psychological treatment response, such as cognitive insight, may underlie differential effectiveness of therapies including Cognitive Behavioural Therapy (CBT) and Metacognitive Training (MCT) [12].

MCT, a psychological intervention derived from CBT, and originally developed by Moritz and Woodward [13], is a promising intervention for individuals with psychotic disorders, particularly schizophrenia. Designed to target cognitive biases, MCT aims to reduce symptom severity without directly confronting delusional content [14]. Importantly, MCT uses normalizing language and examples to encourage participants to discuss and think about their symptoms and experiences with psychosis. Its efficacy has been demonstrated across various clinical settings, with improvements observed in positive, negative, and general symptoms of psychosis [15] and it is strongly recommended (WFSBP-grade 1) to improve positive symptoms [16]. Despite growing evidence supporting MCT’s effectiveness, relatively few studies have examined whether treatment outcomes differ by biological sex. To date, only one study has explicitly investigated sex differences in response to MCT [12]. This study found that females in the MCT group showed greater improvements in general symptoms, cognitive insight, and reduced personalizing bias and dependency-related beliefs, while males improved more in frustration tolerance and affective reasoning tasks, suggesting the existence of sex-specific mechanisms underlying response to MCT. However, this study was limited to a sample of participants with first episode psychosis. It therefore remains unclear whether these sex-related differences, particularly in symptom outcomes, generalize across MCT formats and different stages of the disorder.

The current study addresses this gap by analyzing harmonized retrospective data from the PERMEPSY project (www.permepsy.org), which compiled pre- and post-treatment assessments from international studies involving different versions of MCT on participants at various stages of the disease. Using psychotic symptoms as the primary outcome measure, we investigated whether biological sex moderates symptom trajectories following MCT. By leveraging a large, harmonized dataset and standardized analytic procedures, this study provides a robust examination of sex-specific treatment effects in psychosis. Specifically, the aims of this study are to provide further evidence of the effect of MCT at reducing psychotic symptoms and explore whether the recovery trajectory is sex-specific. We hypothesized a reduction of psychotic symptoms in both males and females, with a more pronounced reduction in general symptoms among females, in line with the findings reported by Salas-Sender et al. [12].

## Methods

### Data source and participants

This study utilized data from the retrospective harmonized database developed within the framework of the European ERAPERMED project “Towards a Personalized Medicine Approach to Psychological Treatment of Psychosis” (PERMEPSY). The dataset comprises information from 698 patients diagnosed with psychotic disorders who received MCT across 22 international studies. The harmonized dataset includes pre- and post-treatment assessments of clinical symptoms, as well as sociodemographic variables such as age, biological sex, educational background, living situation and employment status.

Participants included in the harmonized database were required to meet the following criteria: (1) age ≥ 16 years; (2) a diagnosis of schizophrenia spectrum disorder; and (3) symptom onset within the past five years.

Exclusion criteria were: (1) the presence of severe organic brain dysfunction or intellectual disability; and (2) prior participation in MCT interventions.

The harmonization process followed a structured, multi-step approach involving: (1) selection of relevant datasets; (2) identification and mapping of variables of interest; (3) transformation of variables into a common format; and (4) data cleaning. Variables were harmonised using Python scripts in Jupyter Notebooks, ensuring reproducibility and transparency. Psychological indicators were recalculated where possible to ensure consistency across studies. For example, Positive and Negative Syndrome Scale (PANSS; [17]) scores were recalculated from item-level data when available. Missing or invalid values were coded as “NA”. The harmonised dataset includes 563 variables, covering both pre- and post-treatment assessments. A detailed account of the harmonisation procedure, including data distribution, can be found in König et al. [18].

For the present analysis, participants were included if they had completed at least one PANSS subscale (positive, negative, general symptoms, or total score) at pre-treatment assessments, resulting in a final sample of 573 participants. For each participant, a single pre-treatment and a single post-treatment PANSS assessment were retained, as these were the most consistently available timepoints across the 22 contributing studies. The time interval between assessments varied across studies and details are provided in Supplementary Table S1 in S1 File.

### Intervention

MCT is a manualized psychological group intervention designed to help patients reflect on their thinking patterns and recognize cognitive biases from a “bird’s-eye view.” Exercises are designed to elicit “aha” moments, enabling participants to better understand their cognitive processes and psychotic experiences. Rather than directly confronting psychotic content (which can be distressing), MCT relies on emotionally neutral exercises to explore underlying cognitive mechanisms. The overarching goals are to help participants identify and modify dysfunctional thinking patterns, reduce symptom intensity, and prevent relapse.

MCT was developed to target cognitive biases that contribute to the maintenance of psychotic symptoms, particularly delusions and hallucinations. The standard MCT program consists of 8–10 modules addressing biases such as jumping to conclusions, attributional style, belief inflexibility, and overconfidence in errors. Some modules also focus on affective symptoms, stigma, and self-esteem. MCT is typically delivered in group format but can also be adapted for individual sessions (MCT+). Multiple variants of MCT are available and freely accessible via the official website (http://uke.de/mct).

Within the PERMEPSY harmonized dataset, patients received different MCT variants depending on the original study protocol and clinical context. These included the original group MCT, MCT+ (one-to-one module sessions), and MCT-N, a variant adapted to address negative symptoms. Despite differences in delivery format and module emphasis, all versions share a common therapeutic framework aimed at enhancing metacognitive awareness and promoting cognitive insight.

### Materials

The present study utilized a harmonized dataset comprising sociodemographic and psychological variables collected across 22 international studies involving patients who received MCT for psychosis. Sociodemographic information was standardized across sources and included key indicators of personal and contextual background based on availability across datasets. Psychological measures were harmonised to ensure consistency in scoring and interpretation across studies. A comprehensive description of the available variables in the harmonised dataset can be found in König et al. [18].

The following variables from the harmonized dataset were included in the analyses:

- Sociodemographic variables included age (in years), biological sex (male or female), educational background (years of basic and further education), living situation (e.g., living alone, with family, in a residence), diagnosis (DSM-5) and employment status (e.g., active, inactive, student, retired, disabled). Biological sex was the primary variable of interest. All sociodemographic data were collected at pre-treatment only
- Positive and Negative Syndrome Scale (PANSS; [17]) score were used to asses psychotic symptoms severity. The PANSS comprises three subscales: positive symptoms (PANSS_P; 7 items), negative symptoms (PANSS_N; 7 items) and General psychopathology (PANSS_G; 16 items), yielding a total score across 30 items (PANSS_T). All items are rated on a 7-point Likert scale, with PANSS_P and PANSS_N ranging from 7 to 49, PANSS_G scores from 16 to 112 and PANSS_T scores from 30 to 210.

### Data analysis

Statistical analyses were conducted to examine the effects of MCT on clinical outcomes, with a particular focus on biological sex differences in treatment response. Prior to primary analyses, baseline equivalence between male and female participants was assessed using Mann-Whitney U tests for continuous variables and chi-square tests for categorical variables. The primary analytical approach consisted of a series of repeated-measures analyses of variance (ANOVAs), performed separately for each psychological variable. Each model tested the main effect of Time (pre- vs. post-treatment), Sex, and the Time × Sex interaction. Estimated marginal means and 95% confidence intervals by sex and time point are reported in Supplementary Table S2 in S1 File.

Prior to conducting ANOVAs, assumptions of normality and homogeneity of variance were evaluated. Shapiro–Wilk tests indicated significant deviations from normality across most variables; however, given the large sample sizes (n > 403 for all analyses), ANOVA was deemed robust to this violation provided that homogeneity of variance was met [19]. Homogeneity of variance was assessed using Levene’s tests. Although one outcome variable violated this assumption (PANSS_N; pre-treatment: *F* = 7.22, *p* = 0.008; post-treatment: *F* = 6.72, *p* = 0.010), analyses were nonetheless conducted, and results for this outcome should be interpreted with caution.

To assess the robustness of primary findings in a clinically relevant subgroup, a pre-specified sensitivity analysis was conducted restricting the sample to participants with a FEP diagnosis. This subgroup was selected given that prior studies reporting sex-specific MCT effects were exclusively conducted in FEP populations [12]. The same repeated-measures ANOVA procedure was applied, and results are reported in Supplementary Table S3 in S1 File.

Missing data patterns were examined by testing whether biological sex was associated with post-treatment data availability for each PANSS subscale, using chi-square tests of independence. Results are reported in Supplementary Table S4 in S1 File. No significant association was found for any subscale, supporting the validity of complete case analyses under a Missing at Random assumption.

All analyses were conducted using Jamovi statistical software. For each model, we report partial eta-squared (*η^2^p*) as the effect size measure for the main and interaction effects, together with 90% confidence intervals computed using the non-central F-distribution method [20] implemented in the effectsize R package [21]. Ninety percent, rather than 95%, confidence intervals were used for *η^2^p* because F-tests are inherently one-sided, meaning that a 95% CI can include zero even when the corresponding omnibus test is statistically significant [20,22]. For comparison with effect sizes reported in prior meta-analytic literature on MCT, within-subject standardized effect sizes (Cohen’s *d*_z_) were additionally computed for the main effect of Time on each PANSS subscale [23]. All statistical tests were two-tailed, with significance set at *p* < .05.

### Ethics statement

Ethical approval for the reuse of anonymized retrospective data in the analysis was obtained from the relevant institutional review boards at each participating center. Specifically, approval was granted by the Research Ethics Committee of Fundació Sant Joan de Déu on 27 April 2023 (approval code PIC-68-23), the Lokale Psychologische Ethikkommission at the Zentrum für Psychosoziale Medizin of UKE on 29 March 2023 (approval code LPEK-0603), the Comité Ético Científico of the Servicio de Salud Valparaíso San Antonio on 27 September 2023 (approval code N°54/2023), and the Ethics Committee of the Universitat Politècnica de Catalunya (UPC) on 11 December 2023 (approval code 2023.13). All data were anonymized prior to harmonization. All studies included in the retrospective dataset received ethical approval from the relevant ethics committees and informed consent was obtained in accordance with local regulations. The studies and the project leading the harmonisation process followed the ethical principles of the Declaration of Helsinki code in its latest version.

## Results

A total of 573 individuals with SSD were included in the analysis. Participant age ranged from 16 to 68 years (*M* = 34.7, *SD* = 12.3), and participants had completed an average of 11.5 years of education (*SD* = 3.08). Sex distribution comprised 358 males (62.5%) and 215 females (37.5%). Living situation analysis revealed that most participants (81.8%) lived with family members or parents, while a small minority (3.6%) resided alone. Results indicated that 40.9% of patients were inactive or unemployed, 19.0% were actively employed, and 15.9% on temporary sick leave. Length of illness had a mean duration of 5.28 years (*SD* = 8.14, median = 2.0 years). Diagnostic distribution indicated that most participants were diagnosed with schizophrenia, accounting for 56.0% of the sample, while 9.2% were diagnosed with schizoaffective disorder, 8.9% with first episode psychosis, and 25.9% with other psychotic spectrum disorders Baseline comparisons between male and female participants revealed significant differences in age (*p* < .001), positive symptoms (*p* = .014), negative symptoms (*p* = .006), and diagnostic distribution (*χ^2^* = 21.24, *p* = .002, *V* = .19), with males being younger and presenting with more severe positive and negative symptoms at baseline. No significant differences were found for education, illness duration, or general psychopathology (all *p* > .10). Full baseline characteristics by sex are presented in Table 1. The distribution of PANSS scores at pre- and post-treatment is presented in Table 2.

**Table 1:** Sociodemographic and clinical characteristics of the retrospective sample.

	Male	Female	Total	*p*
**Biological Sex**	358 (62.5%)	215 (37.5%)	573	
**Age (in years)**	32.9 (11.6)	37.7 (12.9)	34.7 (12.3)	<.001
**Education (in years)**	11.4 (3.12)	11.8 (2.98)	11.5 (3.08)	.102
**Illness Duration (in years)**	4.81 (7.84)	6.12 (8.61)	5.28 (8.14)	.177
**Diagnosis**				.002
Schizophrenia	197 (55.0%)	124 (57.7%)	321	
Other SSD	75 (20.9%)	39 (18.1%)	114	
Schizoaffective Disorder	26 (7.3%)	27 (12.6%)	53	
FEP	34 (9.5%)	17 (7.9%)	51	
Delusional Disorder	13 (3.6%)	6 (2.8%)	19	
Brief Psychotic Disorder	7 (2.0%)	2 (0.9%)	9	
Schizophreniform Disorder	6 (1.7%)	0 (0.0%)	6	

SSD = Schizophrenia Spectrum Disorder; FEP = First Episode Psychosis; Values represent mean (standard deviation) for continuous variables and *n* (%) for categorical variables. *p*-values derived from Mann-Whitney U tests for continuous variables and chi-square test for Diagnosis; Bold *p*-values indicate statistical significance (*p* < .05).

**Table 2:** Descriptives of sample’s psychotic symptom distribution (pre- and post-treatment).

		Pre				Post		
	Sex	N	Mean (SD)	Range	*p*	N	Mean (SD)	Range
**PANSS_P**	**Male**	349	15.9 (6.66)	7–39	**.014**	302	13.3 (5.85)	7–34
	**Female**	211	14.6 (6.22)	7–37		177	12.5 (5.54)	7–35
**PANSS_N**	**Male**	311	15.3 (6.7)	7–44	**.006**	271	14.2 (6.41)	7–37
	**Female**	186	13.7 (5.43)	7–37		157	12.6 (5.1)	7–27
**PANSS_G**	**Male**	290	31.5 (10.1)	16–67	.346	256	27.6 (9.26)	16–68
	**Female**	169	30.4 (9.46)	16–67		147	26.2 (8.33)	16–52
**PANSS_T**	**Male**	287	62.7 (20.4)	31–144	.057	256	54.8 (18.7)	30–138
	**Female**	168	58.5 (17.7)	30–122		147	50.6 (16.3)	30–100

PANSS_P = Positive symptom subscale of the Positive and Negative Syndrome Scale (PANSS); PANSS_N = Negative symptom subscale of the PANSS; PANSS_G = General symptom subscale of the PANSS; PANSS_T = Total score in the PANSS; *p*-values derived from Mann-Whitney U tests for sex differences at baseline; Bold *p*-values indicate statistical significance (*p* < .05).

Analysis of PANSS_T scores revealed a significant main effect of Time, indicating a reduction in overall symptom severity following treatment (*F*(1, 395) = 139.169, *p* < .001, *η^2^p* = .261, 90% *CI* [.202,.318]). The main effect of sex approached, but did not reach, statistical significance (*F*(1, 395) = 3.60, *p* = .058, *η^2^p* = .009, 90% *CI* [.000,.031]). The time × sex interaction was not significant (*F*(1, 395) = 0.514, *p* = .474, *η^2^p* = .001, 90% *CI* [.000,.014]), suggesting similar therapeutic gains for both male and female participants.

For positive symptoms (PANSS_P), a significant main effect of Time was observed (*F*(1, 476) = 170.347, *p* < .001, *η^2^p* = .264, 90% *CI* [.210,.316]), reflecting reduced positive symptom severity following MCT. The main effect of Sex showed a trend toward significance (*F*(1, 476) = 3.24, *p* = .073, *η^2^p* = .007, 90% *CI* [.000,.024]), but did not reach the conventional threshold for statistical significance. The time × sex interaction was not significant (*F*(1, 476) = 0.323, *p* = .570, *η^2^p* = .001, 90% *CI* [.000,.010]), indicating equivalent treatment-related changes across sexes.

Analysis of negative symptoms (PANSS_N) revealed a significant main effect of Time (*F*(1, 424) = 27.205, *p* < .001, *η^2^p* = .060, 90% *CI* [.029,.101]), indicating a reduction in negative symptom severity following treatment. A significant main effect of Sex was also observed (*F*(1, 424) = 7.28, *p* = .007, *η^2^* = .017, 90% *CI* [.003,.043]), with females exhibiting lower levels of negative symptoms overall. The Time × Sex interaction was not significant (*F*(1, 424) = 0.166, *p* = .684, *η^2^p* = .000, 90% *CI* [.000,.009]), suggesting that the magnitude of symptom change did not differ between males and females.

For general psychopathology (PANSS-G), a significant main effect of Time was found (*F*(1, 397) = 139.43, *p* < .001, *η^2^p* = .260, 90% *CI* [.202,.317]), reflecting improvement in general symptom severity following treatment. Neither the main effect of Sex (*F*(1, 397) = 1.21, *p* = .273, *η^2^p* = .003, 90% *CI* [.000,.019]) nor the Time × Sex interaction (*F*(1, 397) = 1.02, *p* = .313, *η^2^p* = .003, 90% *CI* [.000,.017]) reached statistical significance, indicating similar treatment responses across sexes.

Results of the pre-specified FEP sensitivity analysis are presented in Supplementary Table S3 in S1 File. Significant Time effects were observed for positive symptoms, negative symptoms, and total score, with general symptoms approaching significance. No significant main effect of Sex and no significant Time × Sex interaction were detected for any PANSS subscale, consistent with the primary analyses. Given the small subsample size, these results should be interpreted as exploratory.

In summary, the results demonstrated that significant reductions in psychotic symptomatology were observed across all measured domains following MCT. Females tended to exhibit lower levels of negative and overall symptoms. Both male and female participants experienced comparable reductions in positive, negative, general, and total psychotic symptoms following MCT, with sex showing no moderating effect on treatment outcomes.

## Discussion

This study aimed to investigate whether biological sex moderates symptom trajectories following Metacognitive Training (MCT) in individuals with SSD, using harmonized retrospective data from the PERMEPSY project. Overall, the findings are consistent with prior evidence of MCT-associated reductions in psychotic symptoms across positive, negative, general, and total psychotic symptoms domains, with significant symptom changes observed following treatment in both male and female participants. Biological sex did not significantly moderate treatment outcomes, suggesting that the therapeutic benefits of MCT are broadly comparable across sexes.

The significant main effect of Time across all PANSS subscales is consistent with previous literature demonstrating the efficacy of MCT in reducing psychotic symptoms severity without directly confronting delusional content [15,24]. While it cannot be ruled out that other factors, such as concurrent treatments or regression to the mean, accounted for some of the observed symptom changes, findings are in line with prior evidence supporting the role of MCT in symptom improvement. Importantly, the absence of significant Time × Sex interactions suggests that both males and females experienced comparable symptom changes from MCT across symptom domains, reinforcing its applicability as a broadly effective psychological intervention.

Interestingly, despite the absence of Time × Sex interaction for any PANSS subscale, a significant main effect of Sex was observed for negative symptoms, with females exhibiting lower levels of negative symptoms overall. The main effect of Sex did not reach statistical significance for positive symptoms, general psychopathology or total score. Baseline comparisons confirmed that males presented with significantly higher positive and negative symptom severity and a distinct diagnostic distribution compared to females (see Table 1), consistent with well-documented sex differences in clinical presentation [2,5]. Importantly, when expressed as standardized mean differences (Cohen’s *d*_z_) comparable to those reported in the MCT literature, the magnitude of symptom change for positive symptoms (*d*_z_ = 0.60) was broadly consistent with, albeit modestly higher than, meta-analytic estimates for positive symptoms (*g* = 0.50, [24]; *g* = 0.47, [15]), and the magnitude for negative symptoms (*d_z_* = 0.25) closely matched meta-analytic estimates for negative symptoms (*g* = 0.23, [24]; *g* = 0.23, [15]. The effect for PANSS total score (*d*_z_ = 0.59) was somewhat larger than the pooled estimate reported for total psychotic symptoms by Meinhart et al. [15] (*g* = 0.39, 95% *CI* [0.25, 0.54]), although the respective confidence intervals showed marginal overlap. No directly comparable meta-analytic estimate for general psychopathology was identified in the MCT literature. Nonetheless, these group-level effect sizes should be interpreted with caution, particularly given the heterogeneous, retrospective nature of the sample. Future studies should complement group-level analyses with responder rates and clinically meaningful change indices to better capture individual-level clinical relevance. Furthermore, although statistically significant symptom reductions were observed, the absence of responder analyses and clinically meaningful change indicators prevents conclusions regarding the clinical importance of these changes at the individual patient level. Nonetheless, the consistency of symptom changes observed across domains and sexes is in line with the growing body of evidence supporting MCT as a transdiagnostic intervention for psychosis.

The lack of significant sex and time interactions contrasts with previous findings from Salas-Sender et al. [12], who reported greater sex-specific improvements for females in general symptoms within a FEP sample. This discrepancy may be explained by differences in sample composition, as the present study included participants at various stages of illness, with substantial variability in illness duration, age, and clinical presentation, as well as exposure to multiple MCT formats. Such heterogeneity may have obscured sex-specific effects that are more readily detectable in more homogeneous samples. Notably, the pre-specified sensitivity analysis restricted to FEP participants also yielded no significant Time × Sex interactions across any PANSS subscale. However, given the limited statistical power of this subgroup, these null findings should be interpreted with caution and do not preclude the existence of stage-specific sex effects. SSD onset and course differ by sex, with males more frequently experiencing onset in early adulthood, whereas females tend to show a later peak in onset during their late twenties, as well as a second peak around menopause, typically after the age of 40 [2,25,26]. Females with later-onset schizophrenia often exhibit fewer premorbid impairments compared to those with early-onset or compared to males [26,27], and generally demonstrate more favorable outcomes during early phases of psychosis [9,10]. However, longitudinal studies indicate that sex-related differences tend to diminish or reverse as illness progresses, with symptom profiles and functional outcomes converging in late-life schizophrenia [3,28]. Taken together, these findings suggest that sex may moderate the manifestation of psychosis over time, in a stage-dependant manner [29]. This dynamic may have limited our ability to detect sex differences in treatment response, as illness stage was not explicitly controlled for in the present analyses. Future research should therefore examine sex effects within more clearly defined clinical subgroups, such as individuals at early stages of illness or those receiving specific MCT formats.

Several limitations of the present study should be acknowledged. First, the retrospective nature of the dataset limits causal inference and introduces variability in assessment procedures. Concurrent treatments such as medication adjustments or other psychosocial interventions could not be controlled for and may have contributed to observed symptom changes. Second, missing data in sociodemographic variables and PANSS subscales may have affected statistical power and generalizability. Third, while the harmonization process ensured consistency across studies, residual heterogeneity in intervention delivery and clinical context may have influenced outcomes. Notably, the absence of study-of-origin identifiers and MCT format variables in the PERMEPSY harmonized database precluded the use of linear mixed-effects models with study as a random effect and sensitivity analyses stratified by MCT variant, meaning that potential clustering effects across the contributing studies could not be formally modelled [18,30]. This limitation reflects not only constraints of the final analytic dataset but also a limitation of the harmonization strategy itself. Future harmonization initiatives should preserve study-level identifiers and intervention-format information to enable more rigorous secondary analyses and better account for sources of between-study heterogeneity. Fourth, our analyses relied on the conventional PANSS subscales, which may not fully represent the five-factor structure originally proposed by Van der Gaag and colleagues [31] and later supported by Shafer and Dazzi [32]. These authors identified Positive, Negative, Disorganization, Affect, and Resistance dimensions that differ from the traditional subscale organization. As a result, the use of conventional subscales may have limited the sensitivity of the analyses to detect changes in specific symptom dimensions, potentially underrepresenting shifts in certain symptom dimensions within the five-factor model. Fifth, the observed baseline imbalances in age, diagnostic distribution, and symptom severity between males and females may have complicated the interpretation of longitudinal symptom trajectories. Differences in initial clinical characteristics could have increased variability in treatment-related change, potentially masking or diluting subtle Time × Sex interactions. Consequently, the absence of significant sex moderation effects should not be interpreted as definitive evidence that sex plays no role in treatment response, but rather as an indication that any such effects were not detectable within the context of the heterogeneous baseline profiles represented in the present sample. Despite these limitations, the study offers valuable insights into the generalizability of MCT across sexes. The findings suggest that symptom reductions following MCT are comparable in males and females, with no evidence of sex-specific symptom trajectories. Our results are consistent with another study from our research team [33], who found no moderating effect of sex on MCT efficacy on cognitive insight and the jumping to conclusions bias in heterogeneous samples, suggesting that sex differences may be more detectable in specific subgroups (e.g., FEP) or outcome domains rather than in overall symptom trajectories. On the other hand, the results contrast previous evidence suggesting sex differences may emerge more clearly in cognitive domains rather than in global symptom severity [34], since MCT was found to enhance specific neurocognitive functions in FEP, showing promising effects for women in verbal memory processes. Overall, these results support the integration of MCT into routine care for psychosis, while highlighting the need for further research into personalized approaches that consider sex and other individual differences.

It is also important to note that although this study examined biological sex (male/female) as recorded in clinical datasets, observed differences may partially reflect gendered experiences and socially constructed roles. Gender, as a social construct, influences help-seeking behavior, stigma, and therapeutic engagement, which may shape symptom presentation and treatment outcomes [35,36]. Social and cultural factors such as gender role expectations and help-seeking behaviors may influence therapeutic engagement and outcomes. For example, some studies suggest women with psychosis are more likely to seek treatment earlier and more frequently than men [37], whereas men may experience feelings of weakness or shame during early help-seeking attempts [38].

Moreover, stigma associated with psychosis appears to be both pervasive and gendered. Men may internalize stigma through societal norms emphasizing emotional suppression and self-reliance, potentially contributing to more pronounced negative symptoms, reduced emotional expressivity, and lower engagement in therapeutic processes [39,40]. Men are also often perceived as more dangerous or emotionally suppressed, which may exacerbate negative symptoms, social rejection and internalized stigma [41,42]. In contrast, women may face stigma related to perceived emotional instability or dependency, which can amplify affective symptoms and contribute to higher general psychopathology [41,42]. Public stigma tends to be particularly severe for individuals exhibiting positive symptoms, especially when presented by men, who are often perceived as more intentional or threatening [41]. These gendered stigma experiences may influence both baseline symptom profiles and perceived treatment gains. In terms of clinical importance, although previous studies have not found statistically significant differences in stigma improvement between males and females [43], different moderators of stigma improvement emerge in each sex, suggesting potential sex differences in stigma trajectories. Finally, of important note, however, the present analyses are limited to biological sex as recorded in clinical datasets, and no direct measures of gender-related constructs were available. Therefore, any discussion of gender-related mechanisms should be considered speculative and not directly supported by the data.

Although MCT’s normalizing and non-confrontational approach may help mitigate stigma-related barriers to engagement, future studies should explicitly incorporate measures of stigma, gender role expectations, and subjective treatment experiences to better understand their interaction with symptom trajectories and therapeutic outcomes. In this context, integrating qualitative methodologies, such as semi-structured interviews or focus groups, would allow for a deeper exploration of patients’ perspectives, expectations, and perceived treatment needs across genders, thereby complementing quantitative findings and advancing more personalized and sex- and gender-sensitive approaches to psychosis care.

## Conclusions

This retrospective analysis of harmonized data from the PERMEPSY project demonstrates significant symptom reductions across positive, negative, general, and total domains following MCT, consistent with prior evidence supporting its broad applicability for psychosis regardless of biological sex.

These findings suggest that while sex-specific clinical profiles may influence baseline symptomatology, they do not appear to significantly alter the trajectory of symptom changes observed following MCT, supporting its use as a transdiagnostic and inclusive psychological intervention. Future research should explore whether more nuanced gender-related factors, such as social roles, stigma, and help-seeking behaviors, might interact with treatment engagement and outcomes. Prospective studies with stratified samples and standardized MCT formats could help clarify these dynamics and inform personalized approaches to care.

## Supporting information

S1 File Supplementary Tables S1–S4. Table S1: Characteristics of the 22 studies included in the PERMEPSY database. Table S2: Estimated marginal means and 95% confidence intervals by biological sex and time point, derived from repeated-measures ANOVA models (full sample). Table S3: FEP Sensitivity Analysis. Table S4: Chi-square tests of independence examining the association between biological sex and post-treatment data availability for each PANSS subscale.
(DOCX)
