# Supplementary material for: Exploring sex-specific symptom trajectories in psychosis following metacognitive training (MCT): Evidence from a large-scale retrospective harmonized database
Source: PLoS One. 2026 Sep 21;21(9):e0356632. doi: 10.1371/journal.pone.0356632 (PMC13592629; doi:10.1371/journal.pone.0356632)
Supplement: Supplementary file 1 [file pone.0356632.s001.docx]

**S1 Table. Characteristics of the 22 studies included in the PERMEPSY database.**

| **Main**  **author** | **DOI** | **Year of publication** | **Country** | **Center** | **Setting** | **Diagnosis** | **Sex**  (♂ - ♀) | **MCT Format** (participants) | **Controls** (participants) | **MCT sessions**  (Max) | **Pre-post interval** |
| --- | --- | --- | --- | --- | --- | --- | --- | --- | --- | --- | --- |
| Acuña | Unpublished | - | Chile | - | Outpatients | SCZ | 29 - 17 | MCT (46) | - | 10 | 10 weeks |
| Balzan | 10.1093/schbul/sby152 | 2019 | Australia | Mono | Outpatients | SSD | 32 - 22 | MCT+ (27) | CR (27) | 4 | 4 weeks |
| Favrod | 10.1016/j.jtcc.2015.06.002 | 2015 | Switzerland | Multi  (2) | Outpatients | SSD | 34 - 18 | MCT (26) | TAU (26) | 8 | 8 weeks |
| Fujii | [10.11596/asiajot.17.45](https://doi.org/10.11596/asiajot.17.45) | 2021 | Japan | Mono | - | SSD | 10 - 7 | MCT (9) | OT (8) | 16 | 4 months |
| Gawęda | Unpublished | - | Poland | - | In and outpatients | NS | 23 - 25 | MCT (25) | Discussion (23) | NA | NA |
| Ishikawa | 10.1016/j.schres.2019.08.006 | 2020 | Japan | Multi  (6) | In and outpatients | SSD | 25 - 25 | MCT (24) | TAU (26) | 10 | 10 weeks |
| Kuokkanen | 10.1002/cbm.1905 | 2014 | Finland | Mono | Inpatients  *(Forensic)* | SCZ | 10 - 0 | MCT (10) | - | 8 | 4 weeks |
| Leanza | 10.1016/j.jbtep.2020.101547 | 2020 | Germany | Mono | In and outpatients | SSD | 51 - 41 | MCT+ (46) | CR (46) | 12 | 6 weeks |
| Lopez-Morinigo | 10.1038/s41537-022-00316-x | 2023 | Spain | Mono | Outpatients | SSD | 41 - 36 | MCT (39) | PE (38) | 10 | NA |
| Moritz 1 | 10.1017/S0033291710002618 | 2011 | Germany | Mono | Inpatients | SCZ | 31 - 17 | MCT+ (24) | CR (24) | 8 | 4 weeks |
| Moritz 2 | 10.1016/j.schres.2013.10.007 | 2013 | Germany | Multi  (2) | In and outpatients | SSD | 97 - 56 | MCT (79) | CR (74) | 8-16 | 4-8 weeks |
| Moritz 3 | 10.1016/j.brat.2010.11.010 | 2011 | Germany | Multi  (2) | Outpatients | SSD | 28 - 8 | MCT (18) | TAU (18) | 8 | 4 weeks |
| Ochoa 1 | 10.1017/S0033291716003421 | 2017 | Spain | Multi  (9) | - | SSD | 85 - 37 | MCT (65) | PE (57) | 8 | 8 weeks |
| Ochoa 2 | NA | 2020 | Spain | Multi  (10) | - | SSD | 44 - 25  (1 NA) | MCT+ (36) | TAU (34) | 10 | 10 weeks |
| Ochoa 3 | Unpublished | - | Spain | - | - | SSD | 28 - 22  (4 NA) | MCT (54) | - | 8-10 | 8-10 weeks |
| De Pinho | 10.1111/jan.14627 | 2020 | Portugal | Multi  (4) | In and outpatients | SCZ | 30 - 22 | MCT (26) | TAU (26) | 8 | 4 weeks |
| Simon-Exposito | 10.3390/ijerph16224541 | 2019 | Spain | Multi  (2) | Inpatients | SSD | 16 - 6 | MCT (11) | TAU (11) | 16 | 8 weeks |
| So | 10.3389/fpsyg.2015.00730 | 2015 | China | Mono | Outpatients | SSD | 24 - 20 | MCT+ (23) | TAU (21) | 4 | 4 weeks |
| Swanson | 10.1002/cpp.2692 | 2021 | Scotland | Mono | In and outpatients | SSD | 13 - 2 | MCT-N (15) | - | 8 | NA |
| Tanoue | Unpublished | - | Japan | - | Outpatients | SCZ | 14 - 8 | MCT (22) | - | NA | NA |
| Ussorio | 10.1111/papt.12059 | 2016 | Italy | Mono | Outpatients  *(Early intervention)* | SSD | 30 - 13 | MCT Young Version (43) | - | 16 | 4 months |
| Yildiz | 10.29399/npa.23095 | 2019 | Turkey | Mono | Outpatients | SSD | 13 - 7 | MCT (10) | PST (10) | 40 | 20 weeks |

MCT = MetaCognitive Training (group); MCT+ = MetaCognitive Training (individual); MCT-N = MetaCognitive Training (group) - variant targeting negative symptoms; CR = Cognitive remediation; OT = Occupational therapy; PE = Psychoeducation; PST = Psychosocial Skills Training; TAU = Treatment as usual; SSD = Schizophrenia spectrum disorder; SCZ = Schizophrenia; NA = Not available.

**S2 Table. Estimated marginal means and 95% confidence intervals by biological sex and time point, derived from repeated-measures ANOVA models (full sample).**

|  | **PANSS_P** |  | **PANSS_N** |  | **PANSS_G** |  | **PANSS_T** |  |
| --- | --- | --- | --- | --- | --- | --- | --- | --- |
|  | **Pre** | **Post** | **Pre** | **Post** | **Pre** | **Post** | **Pre** | **Post** |
| **Male** | 15.99  [15.25–16.74] | 13.35  [12.70–14.00] | 15.20  [14.45–15.95] | 14.23  [13.52–14.94] | 31.02  [29.83–32.21] | 27.57  [26.47–28.67] | 61.68  [59.31–64.05] | 54.76  [52.55–56.97] |
| **Female** | 14.89  [13.91–15.86] | 12.46  [11.62–13.31] | 13.71  [12.72–14.70] | 12.58  [11.64–13.52] | 30.34  [28.77–31.91] | 26.25  [24.79–27.70] | 58.67  [55.55–61.79] | 50.85  [47.94–53.76] |

Values represent estimated marginal means [95% confidence interval] derived from repeated-measures ANOVA models. PANSS-P = Positive symptoms; PANSS-N = Negative symptoms; PANSS-G = General psychopathology; PANSS-T = Total score*.*

**Conclusion**

Inspection of confidence intervals reveals overlapping estimates between male and female participants at both pre- and post-treatment timepoints across all PANSS subscales, with comparable pre-post changes in both groups, suggesting that any potential sex-specific effects are likely too small to be clinically meaningful.

**S3 Table. FEP Sensitivity Analysis**

ST2.1	Descriptive statistics by sex and time point in the FEP subsample

|  | **PANSS_P Pre**  *(n = 50)* | **PANSS_P Post**  *(n = 48)* | **PANSS_N Pre**  *(n = 50)* | **PANSS_N Post**  *(n = 49)* | **PANSS_G Pre**  *(n = 45)* | **PANSS_G Post**  *(n = 45)* | **PANSS_T Pre**  *(n = 45)* | **PANSS_T Pre**  *(n = 45)* |
| --- | --- | --- | --- | --- | --- | --- | --- | --- |
| **Male** | 17.38 (4.36) | 16.67 (3.81) | 17.91 (7.96) | 18.09 (7.77) | 34.71 (6.12) | 34.52 (6.07) | 71.26 (14.32) | 69.84 (13.47) |
| **Female** | 16.31 (5.1) | 15.93 (4.99) | 15.13 (5.32) | 15.93 (5.28) | 34.43 (6.05) | 34.00 (6.29) | 67.21 (12.41) | 66.07 (12.40) |

Values represent mean (standard deviation). PANSS = Positive and Negative Syndrome Scale. PANSS-P = Positive symptoms; PANSS-N = Negative symptoms; PANSS-G = General psychopathology; PANSS-T = Total score.

ST2.2 Repeated-measures ANOVA results for the FEP subsample, examining effects of Time, Biological Sex, and their interaction on PANSS subscales.

|  | **PANSS_P** | | | **PANSS_N** | | | **PANSS_G** | | | **PANSS_T** | | |
| --- | --- | --- | --- | --- | --- | --- | --- | --- | --- | --- | --- | --- |
| **Effect** | **F** | ***p*** | ***η²*** | **F** | ***p*** | ***η²*** | **F** | ***p*** | ***η²*** | **F** | ***p*** | ***η²*** |
| **Time** | 13.07 | **.001** | .009 | 4.58 | **.038** | .001 | 3.63 | .064 | <.001 | 16.47 | **<.001** | .002 |
| **Sex** | 0.32 | .573 | .007 | 1.19 | .280 | .024 | 0.04 | .840 | .001 | 0.82 | .372 | .018 |
| **Time × Sex** | 0.01 | .932 | <.001 | 1.36 | .249 | <.001 | 0.52 | .476 | <.001 | 0.19 | .664 | <.001 |

η² = partial eta-squared. Bold p-values indicate statistical significance (p < .05). PANSS = Positive and Negative Syndrome Scale. PANSS-P = Positive symptoms; PANSS-N = Negative symptoms; PANSS-G = General psychopathology; PANSS-T = Total score.

**Conclusion**

Across all PANSS subscales, significant Time effects were observed for positive symptoms (*p* = .001), negative symptoms (*p* = .038), and total score (*p* < .001), with general symptoms approaching significance (*p* = .064). No significant main effect of Sex was detected for any subscale (all *p* > .280). Critically, no significant Time × Sex interaction was detected for any subscale (all *p* > .249), consistent with the primary analyses.

Given the small size of the FEP subsample (n ≈ 45-50 depending on the subscale), these analyses are underpowered and should be interpreted as exploratory only. Null findings in this subgroup cannot be interpreted as evidence for the absence of sex-specific effects in FEP populations.

**S4 Table. Chi-square tests of independence examining the association between biological sex and post-treatment data availability for each PANSS subscale.**

|  | **PANSS_P** |  | **PANSS_N** |  | **PANSS_G** |  | | | **PANSS_T** | |  |
| --- | --- | --- | --- | --- | --- | --- | --- | --- | --- | --- | --- |
|  | **Available** | **Missing** | **Available** | **Missing** | **Available** | **Missing** | | | **Available** | | **Missing** |
| **Male**  (*n*=358) | 302 | 56 | 271 | 87 | 256 | 102 | | | 256 | | 102 |
| **Female** (*n*=215) | 177 | 38 | 157 | 58 | 147 | 68 | | | 147 | | 68 |
|  | **χ²** | ***p*** | **Cramér’s V** |  |  | |  |  | |  |  |
| **PANSS_P** | 0.404 | .525 | .027 |  |  | |  |  | |  |  |
| **PANSS_N** | 0.509 | .476 | .030 |  |  | |  |  | |  |  |
| **PANSS_G** | 0.633 | .426 | .033 |  |  | |  |  | |  |  |
| **PANSS_T** | 0.633 | .426 | .033 |  |  | |  |  | |  |  |

Missing = 1 indicates absence of post-treatment data; Cramér's V reflects effect size, with values < .10 indicate negligible association; PANSS = Positive and Negative Syndrome Scale. PANSS-P = Positive symptoms; PANSS-N = Negative symptoms; PANSS-G = General psychopathology; PANSS-T = Total score.

**Conclusion**

No significant association was found between biological sex and post-treatment data availability for any PANSS subscale (all *p* > .426, all Cramér's V < .033), indicating that missingness was not differentially distributed across sexes and supporting the validity of complete case analyses under a Missing at Random (MAR) assumption.
