# Supplementary material for: Exploring sex-specific symptom trajectories in psychosis following metacognitive training (MCT): Evidence from a large-scale retrospective harmonized database
Source: PLoS One. 2026 Sep 21;21(9):e0356632. doi: 10.1371/journal.pone.0356632 (PMC13592629; doi:10.1371/journal.pone.0356632)
Supplement: Supplementary file 2 [file pone.0356632.s004.docx]

**Response to Reviewers**

**Manuscript:** *"Exploring Sex-Specific Symptom Trajectories in Psychosis Following Metacognitive Training (MCT): Evidence from a large-scale harmonized database"*

Dear Editors and Reviewers,

We thank our reviewers for their detailed and constructive feedback, which has helped us strengthen the conceptual rationale and methodological transparency of the study, refine our interpretations, and improve the overall quality of the manuscript. We have provided detailed answers to all the issues raised in the revision letter below:

**Journal and Editor Comments**

1. **If the reviewer comments include a recommendation to cite specific previously published works, please review and evaluate these publications to determine whether they are relevant and should be cited. There is no requirement to cite these works unless the editor has indicated otherwise.**

- We thank you for this comment and have taken it into account when revising the manuscript with the reviewer comments.

2. **Please review your reference list to ensure that it is complete and correct. If you have cited papers that have been retracted, please include the rationale for doing so in the manuscript text, or remove these references and replace them with relevant current references. Any changes to the reference list should be mentioned in the rebuttal letter that accompanies your revised manuscript. If you need to cite a retracted article, indicate the article’s retracted status in the References list and also include a citation and full reference for the retraction notice.**

- We thank the editor for this reminder. We have carefully reviewed our reference list and verified all DOIs using the Retraction Watch Database (https://retractiondatabase.org/RetractionSearch.aspx); no retracted articles were identified among our citations. During this verification, we identified three duplicate entries, which have been corrected: references 12 and 17 (Salas-Sender et al., 2020, now reference 12), references 16 and 22 (Goncalves et al., 2025, now reference 16), and references 19 and 28 (König et al., 2025, now reference 18). The reference list has been updated accordingly.

**Reviewer #1**

**Thank you for the careful and thorough revision of the manuscript. The authors have addressed the majority of the concerns raised in the previous review round. In particular, I appreciate the efforts to:**

**1. Remove or substantially reduce causal language throughout the manuscript.
2. Clarify the retrospective and uncontrolled nature of the study.**

**3. Expand discussion of dataset heterogeneity.**

**4. Provide additional information regarding the contributing studies.**

**5. Conduct and report a sensitivity analysis restricted to first-episode psychosis participants.
6. Clarify the distinction between biological sex and gender.**

**7. Improve transparency regarding data availability and governance restrictions.
Overall, the manuscript is now substantially stronger, more transparent, and better aligned with the conclusions supported by the data.**

**I have only a few remaining comments.**

- We sincerely thank the reviewer for the careful evaluation of our revised manuscript and for recognizing the extensive efforts made to address the concerns raised during the previous review round. We greatly appreciate the positive assessment that the manuscript is now stronger, more transparent, and better aligned with the conclusions supported by the data. We are grateful for the reviewer’s constructive feedback throughout the review process, which has significantly contributed to improving the scientific rigor, clarity, and transparency of this work. We have carefully considered the remaining comments and have addressed each of them in detail below.

**1. The authors explain that study-of-origin identifiers and MCT format information were not retained during harmonization, making multilevel modelling impossible. I appreciate this clarification. However, this is not merely a statistical limitation but also a limitation of the harmonization strategy itself. The current wording may leave readers with the impression that mixed-effects modelling was impossible due to the dataset structure, whereas in fact the harmonization process eliminated variables that would have allowed modelling study-level clustering and intervention-format effects. I would recommend slightly strengthening the wording in the limitations section to explicitly acknowledge that future harmonization initiatives should preserve study-level identifiers and intervention-format information to permit more rigorous secondary analyses.**

- We thank the reviewer for this valuable observation. We agree that the inability to model study-level clustering and intervention-format effects reflects not only a statistical limitation of the final dataset but also a limitation of the harmonization strategy adopted during database construction. We have revised the limitations section to clarify that study-of-origin identifiers and MCT format information were not retained during harmonization, which prevented the application of multilevel modelling and stratified analyses by intervention format. We further acknowledge that future harmonization initiatives should preserve these variables to facilitate more rigorous secondary analyses, including the modelling of study-level clustering and intervention-specific effects.

Specifically, we have added the following sentence in page 19: “*This limitation reflects not only constraints of the final analytic dataset but also a limitation of the harmonization strategy itself. Future harmonization initiatives should preserve study-level identifiers and intervention-format information to enable more rigorous secondary analyses and better account for sources of between-study heterogeneity.”*

**2. The newly added baseline analyses indicate significant differences between males and females in age, diagnostic distribution, positive symptoms, and negative symptoms. These differences are important because they complicate interpretation of subsequent symptom trajectories. Although the discussion now mentions these findings, I would encourage the authors to elaborate slightly further on whether such baseline imbalances could potentially mask or dilute sex-by-time interactions. I am not requesting additional analyses, merely a somewhat more explicit acknowledgement of this interpretive limitation.**

- We thank the reviewer for this insightful comment. We have expanded the Limitations Discussion section to explicitly recognize that baseline imbalances between males and females may have reduced our ability to detect sex-by-time interactions. In particular, differences in initial symptom severity and diagnostic composition may have introduced additional variability in change scores, potentially masking or diluting subtle sex-specific treatment effects. We have added this consideration as an important interpretive limitation of the study.

Page 20: “Fifth, the observed baseline imbalances in age, diagnostic distribution, and symptom severity between males and females may have complicated the interpretation of longitudinal symptom trajectories. Differences in initial clinical characteristics could have increased variability in treatment-related change, potentially masking or diluting subtle Time × Sex interactions. Consequently, the absence of significant sex moderation effects should not be interpreted as definitive evidence that sex plays no role in treatment response, but rather as an indication that any such effects were not detectable within the context of the heterogeneous baseline profiles represented in the present sample.*”*

**3. I appreciate the additional discussion regarding statistical versus clinical significance. However, the manuscript still relies almost exclusively on group-level statistical effects. Because responder analyses and clinically meaningful change indices could not be calculated, I suggest adding one sentence explicitly stating that the clinical importance of the observed symptom reductions cannot be determined from the current dataset. This would further align interpretation with the available evidence.**

- We thank the reviewer for this valuable suggestion. We have added a sentence to explicitly acknowledge this limitation and to ensure that our interpretation remains closely aligned with the evidence available from the current dataset.

This sentence can be found in page 18: “*Furthermore, although statistically significant symptom reductions were observed, the absence of responder analyses and clinically meaningful change indicators prevents conclusions regarding the clinical importance of these changes at the individual patient level.”*

**4. The revised data availability section is substantially improved and provides a clearer justification for restricted access. I appreciate the authors’ efforts to provide access procedures, analysis scripts, harmonization pipeline documentation, and OSF materials. I have no further concerns regarding transparency from a scientific perspective, although final evaluation of compliance with PLOS ONE data-sharing policies should remain an editorial decision.**

- We thank the reviewer for this positive assessment of the revised Data Availability Statement. We agree that the final evaluation of compliance with PLOS ONE data-sharing policies falls within the remit of the editorial team, and we remain available to provide any additional information or clarification that may be required by the editors.

We sincerely thank the reviewer for the thoughtful, constructive, and rigorous feedback provided throughout the review process. We greatly appreciate the time and effort invested in evaluating our work and helping us improve the manuscript.

**Reviewer #2**

**5. In my opinion, the authors have made an important effort to improve the original submission, resulting in a ready-to-publish manuscript.**

- We sincerely thank the reviewer for the positive assessment of our manuscript. We greatly appreciate the recognition of the efforts made to address the reviewers’ comments and improve the quality of the original submission. We are pleased that the revisions have strengthened the manuscript and that, in the reviewer's opinion, it is now suitable for publication. We are grateful for the time and consideration dedicated to the evaluation of our work and for the constructive feedback provided throughout the review process.

**Reviewer #3**

**Overall, the manuscript has been substantially improved. The causal language has been toned down, the analytical approach is explained and justified more clearly, the corresponding limitations have been addressed, and a helpful overview over the different included studies has been provided. manuscript.**

- We sincerely thank the reviewer for their evaluation of our revised manuscript. We greatly appreciate the recognition that the revisions have improved the clarity, transparency, and overall rigor of the study. We are also grateful for the reviewer’s continued careful evaluation of the manuscript and for identifying the remaining areas that require clarification. We have carefully considered the comments and we address these points in detail below.

**6. However, there is one section in the discussion that appears a bit unclear and somewhat self-contradictory. This is also related to the fact that the authors seem to have misunderstood my previous comments about confidence intervals for effect sizes. For clarification: In the previous review, I suggested adding confidence intervals to the obtained effect sizes, i.e. main and interaction effects), as this would provide more meaningful information than a post-hoc power analysis. I appreciate the authors’ responsiveness to this comment, and the newly added table of Estimated Marginal Means and 95% confidence intervals is an illustrative and informative addition to the manuscript. However, providing confidence intervals simply for the individual cells (time x sex for each PANSS subscale) does not address the original issue. The here provided cell-level confidence intervals indicate the precision of group scores at a single time point, but do not reflect the precision or uncertainty of the actual main effects and interaction effects derived from the repeated-measures ANOVA models. Instead, the authors may want to look into ways to calculate confidence intervals for the reported partial eta-squared estimates. (see, e.g., for an R-based approach: Guide to Effect Sizes and Confidence Intervals, Jané et al., 2024, https://osf.io/d8c4g/; though other approaches are possible, of course).
.... Side note: I also noticed that the degrees of freedom are missing for the reported F-statistics throughout the manuscript. These should be added, as it is currently unclear exactly how many participants were included in each respective analysis (given different proportions of missing data pre- and post-treatment).**

- We thank the reviewer for this important clarification, and apologize for the misunderstanding regarding the nature of the requested confidence intervals in the previous revision round. We have now computed 90% confidence intervals for the partial eta-squared (η²p) values associated with each main and interaction effect from the repeated-measures ANOVA models, rather than for the estimated marginal means alone. These confidence intervals were computed using the non-central F-distribution method (Steiger, 2004), implemented in the effectsize R package (Ben-Shachar et al., 2020), following the approach recommended in the guide cited by the reviewer (Jané et al., 2024). As recommended in that same guide, we opted for 90%, rather than 95%, confidence intervals for η²p, given that F-tests are inherently one-sided and a 95% CI can therefore include zero even when the corresponding omnibus test is statistically significant. We have also added the degrees of freedom for all reported F-statistics throughout the Results section, addressing the reviewer's side note regarding the number of participants included in each analysis.

*These changes are reflected in the Results section and in the Methods section (page 12): For each model, we report partial eta-squared (η²p) as the effect size measure for the main and interaction effects, together with 90% confidence intervals computed using the non-central F-distribution method, implemented in the effectsize R package. Ninety percent, rather than 95%, confidence intervals were used for η²p because F-tests are inherently one-sided, meaning that a 95% CI can include zero even when the corresponding omnibus test is statistically significant”*

- Additionally, while cross-checking these values against the original jamovi output during this revision, we identified and corrected a transcription error in the reported F-statistic for the Time × Sex interaction on PANSS_T (previously reported as F = 0.474; corrected to F = 0.514).

**Furthermore, the discussion paragraph where the confidence intervals are mentioned is currently written in a somewhat confusing and contradictory manner, making it hard to follow: from p. 19, line 403 (‘Interestingly, while no significant sex ….’) to p. 20, line 418/419 (‘…did not reach statistical significance.’). Here, the authors alternate between stating significant differences, then stating that overlapping CIs show there are no differences, and then pointing to non-significant 'trends' to suggest there might be a difference. This section needs to be streamlined**

- We thank the reviewer for this comment and agree that this paragraph required streamlining. We have substantially revised it to remove the alternating and potentially contradictory reasoning identified by the reviewer. Specifically, we no longer rely on the overlap of confidence intervals around estimated marginal means (Supplementary Table S2) to infer the absence of a sex-specific effect, nor do we refer to non-significant "trends" to suggest a possible effect in the opposite direction. Instead, the revised paragraph reports the pattern of significance for the main effect of Sex and the Time × Sex interaction in a single, non-repetitive statement, and separately situates the magnitude of the Time effect relative to standardized effect sizes (Cohen's dz) reported in prior MCT meta-analyses, which we consider a more informative and directly comparable basis for interpretation.

The revised paragraph now reads (page 17-18): “Interestingly, despite the absence of Time × Sex interaction for any PANSS subscale, a significant main effect of Sex was observed for negative symptoms, with females exhibiting lower levels of negative symptoms overall. The main effect of Sex did not reach statistical significance for positive symptoms, general psychopathology or total score. Baseline comparisons confirmed that males presented with significantly higher positive and negative symptom severity and a distinct diagnostic distribution compared to females (see Table 1), consistent with well-documented sex differences in clinical presentation [2,5]. Importantly, when expressed as standardized mean differences (Cohen's dz) comparable to those reported in the MCT literature, the magnitude of symptom change for positive symptoms (dz = 0.60) was broadly consistent with, albeit modestly higher than, meta-analytic estimates for positive symptoms (g = 0.50, Penney et al. [21]; g = 0.47, Meinhart et al. [15]), and the magnitude for negative symptoms (dz = 0.25) closely matched meta-analytic estimates for negative symptoms (g = 0.23, Penney et al. [21]; g = 0.23, Meinhart et al. [15]). The effect for PANSS total score (dz = 0.59) was somewhat larger than the pooled estimate reported for total psychotic symptoms by Meinhart et al. [15] (g = 0.39, 95% CI [0.25, 0.54]), although the respective confidence intervals showed marginal overlap. No directly comparable meta-analytic estimate for general psychopathology was identified in the MCT literature. Nonetheless, these group-level effect sizes should be interpreted with caution, particularly given the heterogeneous, retrospective nature of the sample. Future studies should complement group-level analyses with responder rates and clinically meaningful change indices to better capture individual-level clinical relevance.”

In conclusion, we would like to express our sincere gratitude to the Editor and all Reviewers for their time, expertise, and constructive feedback throughout the review process. Their thoughtful comments and suggestions have been invaluable in strengthening the scientific rigor, clarity, and overall quality of this manuscript. We greatly appreciate the opportunity to revise our work and believe that the changes implemented in response to the reviewers’ recommendations have substantially improved the paper. We hope that the revised manuscript is now suitable for publication, and we remain available to provide any additional information or clarification if required.
