# Supplementary material for: Exploring sex-specific symptom trajectories in psychosis following metacognitive training (MCT): Evidence from a large-scale retrospective harmonized database
Source: PLoS One. 2026 Sep 21;21(9):e0356632. doi: 10.1371/journal.pone.0356632 (PMC13592629; doi:10.1371/journal.pone.0356632)
Supplement: Peer review history

# Peer review: Decision Letter 0

**DOI:** 10.1371/journal.pone.0356632.r001

**Authors:** César González-Blanch

27 May 2026

Exploring Sex-Specific Symptom Trajectories in Psychosis Following Metacognitive Training (MCT): evidence from a large-scale harmonized database.

PLOS One

Dear Dr. Lamarca,

Thank you for submitting your manuscript to PLOS ONE. After careful consideration, we feel that it has merit but does not fully meet PLOS ONE’s publication criteria as it currently stands. Therefore, we invite you to submit a revised version of the manuscript that addresses the points raised during the review process.

Please submit your revised manuscript by Jul 11 2026 11:59PM. If you will need more time than this to complete your revisions, please reply to this message or contact the journal office at plosone@plos.org. When you're ready to submit your revision, log on to https://www.editorialmanager.com/pone/ and select the 'Submissions Needing Revision' folder to locate your manuscript file.

Please include the following items when submitting your revised manuscript:

- A letter that responds to each point raised by the academic editor and reviewer(s). You should upload this letter as a separate file labeled 'Response to Reviewers'.
- A marked-up copy of your manuscript that highlights changes made to the original version. You should upload this as a separate file labeled 'Revised Manuscript with Track Changes'.
- An unmarked version of your revised paper without tracked changes. You should upload this as a separate file labeled 'Manuscript'

If you would like to make changes to your financial disclosure, please include your updated statement in your cover letter. Guidelines for resubmitting your figure files are available below the reviewer comments at the end of this letter.

If applicable, we recommend that you deposit your laboratory protocols in protocols.io to enhance the reproducibility of your results. Protocols.io assigns your protocol its own identifier (DOI) so that it can be cited independently in the future. For instructions see: https://journals.plos.org/plosone/s/submission-guidelines#loc-laboratory-protocols. Additionally, PLOS ONE offers an option for publishing peer-reviewed Lab Protocol articles, which describe protocols hosted on protocols.io. Read more information on sharing protocols at https://plos.org/protocols?utm_medium=editorial-email&utm_source=authorletters&utm_campaign=protocols.

As the corresponding author, your ORCID iD is verified in the submission system and will appear in the published article. PLOS supports the use of ORCID, and we encourage all coauthors to register for an ORCID iD and use it as well. Please encourage your coauthors to verify their ORCID iD within the submission system before final acceptance, as unverified ORCID iDs will not appear in the published article. *Only*  the individual author can complete the verification step; PLOS staff *cannot*  verify ORCID iDs on behalf of authors.

We look forward to receiving your revised manuscript.

Kind regards,

César González-Blanch, PhD

Academic Editor

PLOS One

Journal Requirements:

When submitting your revision, we need you to address these additional requirements.

1.Please ensure that your manuscript meets PLOS ONE's style requirements, including those for file naming. The PLOS ONE style templates can be found at

https://journals.plos.org/plosone/s/file?id=wjVg/PLOSOne_formatting_sample_main_body.pdf and

https://journals.plos.org/plosone/s/file?id=ba62/PLOSOne_formatting_sample_title_authors_affiliations.pdf

2. Thank you for stating the following in the Competing Interests section:

“I have read the journal's policy and the authors of this manuscript have the following competing interests: Steffen Moritz, one of the co-authors, is the main developer of Metacognitive Training (MCT) for psychosis. To minimize potential bias, he was not involved in data extraction, data harmonization, or statistical analyses for this study. No other authors have competing interests.”

Please confirm that this does not alter your adherence to all PLOS ONE policies on sharing data and materials, by including the following statement: "This does not alter our adherence to  PLOS ONE policies on sharing data and materials.” (as detailed online in our guide for authors http://journals.plos.org/plosone/s/competing-interests).  If there are restrictions on sharing of data and/or materials, please state these. Please note that we cannot proceed with consideration of your article until this information has been declared.

Please include your updated Competing Interests statement in your cover letter; we will change the online submission form on your behalf.

3. Please note that funding information should not appear in any section or other areas of your manuscript. We will only publish funding information present in the Funding Statement section of the online submission form. Please remove any funding-related text from the manuscript.

4. We note that you have indicated that there are restrictions to data sharing for this study. PLOS only allows data to be available upon request if there are legal or ethical restrictions on sharing data publicly. For more information on unacceptable data access restrictions, please see http://journals.plos.org/plosone/s/data-availability#loc-unacceptable-data-access-restrictions.

Before we proceed with your manuscript, please address the following prompts:

a) If there are ethical or legal restrictions on sharing a de-identified data set, please explain them in detail (e.g., data contain potentially identifying or sensitive patient information, data are owned by a third-party organization, etc.) and who has imposed them (e.g., a Research Ethics Committee or Institutional Review Board, etc.). Please also provide contact information for a data access committee, ethics committee, or other institutional body to which data requests may be sent.

b) If there are no restrictions, please upload the minimal anonymized data set necessary to replicate your study findings to a stable, public repository and provide us with the relevant URLs, DOIs, or accession numbers. For a list of recommended repositories, please see

https://journals.plos.org/plosone/s/recommended-repositories. You also have the option of uploading the data as Supporting Information files, but we would recommend depositing data directly to a data repository if possible.

We will update your Data Availability statement on your behalf to reflect the information you provide.

5. In the online submission form you indicate that your data is not available for proprietary reasons and have provided a contact point for accessing this data. Please note that your current contact point is a co-author on this manuscript. According to our Data Policy, the contact point must not be an author on the manuscript and must be an institutional contact, ideally not an individual. Please revise your data statement to a non-author institutional point of contact, such as a data access or ethics committee, and send this to us via return email. Please also include contact information for the third party organization, and please include the full citation of where the data can be found.

6. If the reviewer comments include a recommendation to cite specific previously published works, please review and evaluate these publications to determine whether they are relevant and should be cited. There is no requirement to cite these works unless the editor has indicated otherwise.

Additional Editor Comments:

The revisions mainly concern the analytical approach, interpretation of findings, and handling of heterogeneity across studies. In particular, the reviewers requested either a re-analysis using linear mixed-effects models or a stronger justification for the current analytical strategy, as well as a more cautious interpretation of symptom changes given the absence of a control group. Additional recommended revisions include expanding methodological details regarding the included studies and MCT variants, clarifying the handling of missing data, reporting confidence intervals and effect sizes more systematically, and integrating recent related literature into the discussion.

[Note: HTML markup is below. Please do not edit.]

Reviewer's Responses to Questions

**Comments to the Author**

1. Is the manuscript technically sound, and do the data support the conclusions?

Reviewer #1: Yes

Reviewer #2: Yes

Reviewer #3: Partly

**********

2. Has the statistical analysis been performed appropriately and rigorously?

Reviewer #1: Yes

Reviewer #2: Yes

Reviewer #3: Yes

**********

3. Have the authors made all data underlying the findings in their manuscript fully available?

The PLOS Data policy

Reviewer #1: No

Reviewer #2: No

Reviewer #3: No

**********

4. Is the manuscript presented in an intelligible fashion and written in standard English?

Reviewer #1: Yes

Reviewer #2: Yes

Reviewer #3: Yes

**********

Reviewer #1: This manuscript examines whether biological sex moderates symptom trajectories following Metacognitive Training (MCT) in individuals with schizophrenia spectrum disorders, using a large harmonized retrospective dataset (n = 573) derived from the PERMEPSY consortium.

The study addresses a relevant and timely question, given increasing interest in sex differences in psychosis and the dissemination of scalable psychological interventions. The use of a large international dataset and standardized outcome measures (PANSS) represents a clear strength. The manuscript is generally well structured and written in clear scientific English.

However, several important issues limit the strength of the conclusions. These relate primarily to design limitations, analytic strategy, handling of heterogeneity, and interpretation of findings. While the study meets several PLOS One criteria (originality, clarity), further clarification and methodological strengthening are needed to ensure that conclusions are fully supported by the data.

Major Comments

1. Lack of Control Condition and Causal Interpretation. The most important limitation concerns the absence of a control group. The manuscript repeatedly refers to MCT as "effective" in reducing symptoms. However, the design is retrospective and uncontrolled, meaning that symptom reduction cannot be attributed specifically to MCT. This is particularly relevant given naturalistic symptom fluctuation, regression to the mean, and concurrent treatments (e.g., medication, other psychosocial interventions).

The authors should substantially tone down causal language and reframe findings as "symptom changes observed following MCT exposure" rather than evidence of effectiveness.

2. Heterogeneity of the Dataset (Major Threat to Internal Validity). The PERMEPSY dataset combines 22 studies, multiple countries, different illness stages, and multiple MCT formats (group, MCT+, MCT-N). This introduces substantial clinical and methodological heterogeneity, which is not adequately controlled. Key concerns:

- No modelling of study-level clustering effects

- No adjustment for MCT format differences

- No control for illness stage, duration, or baseline severity

- No multilevel modelling despite nested data structure

At minimum, the authors should acknowledge this as a major limitation more explicitly, and consider including study as a random factor (if possible), or sensitivity analyses (e.g., restricting to subsamples).

3. Analytical Strategy: Limitations of Repeated-Measures ANOVA. The exclusive use of repeated-measures ANOVA is a limitation given the data structure. Issues:

- Missing data is handled implicitly (listwise deletion), leading to varying sample sizes across analyses (as seen in Tables)

- No modelling of individual variability

- No capacity to handle nested or unbalanced data optimally

The authors should justify more explicitly why linear mixed-effects models were not used, or provide a sensitivity analysis using a more robust approach.

4. Interpretation of Non-Significant Sex Effects. The manuscript concludes that "biological sex did not moderate symptom trajectories". However, this conclusion requires caution:

- Effect sizes are very small (η² < .04)

- Non-significant interactions ≠ evidence of absence

- Post hoc power analysis (based on observed effects) is not sufficient to support null findings

Please, reframe conclusions more cautiously: Emphasize lack of detected differences, not absence of true differences, and avoid overreliance on post hoc power.

5. Clinical Significance vs Statistical Significance. Although results are statistically significant, effect sizes are consistently small (η² ≈ .03–.04). The manuscript acknowledges this, but does not fully explore implications. Please, discuss whether observed PANSS changes are clinically meaningful, and consider reporting % of patients achieving clinically relevant improvement or standardized effect sizes (e.g., Cohen’s d).

6. Data Availability and PLOS One Policy. The manuscript states that data cannot be publicly shared due to consortium restrictions. This is potentially in tension with PLOS One’s data availability policy, which requires full availability unless justified. Please, clarify exact restrictions, access procedure, and whether a partial dataset or synthetic dataset could be shared. This point is critical for editorial decision-making.

7. Conceptual Ambiguity: Sex vs Gender. The manuscript includes a discussion on gender-related factors but analyses are strictly based on binary biological sex, and gender-related variables are not measured This creates a conceptual mismatch. Please, clarify distinction more explicitly, and avoid implying conclusions about gender processes without data.

Minor Comments

- Some minor language issues (e.g., “Somes modules”, “adress”) should be corrected.

- Tables are informative but could be streamlined (e.g., reducing missingness columns or improving readability).

- The description of harmonization is useful but could benefit from a brief schematic or summary table.

- Consider reporting confidence intervals systematically.

Conclusion: The manuscript addresses an important question and uses a valuable dataset. However, significant methodological and interpretative issues must be addressed before the conclusions can be considered sufficiently supported.

Reviewer #2: Manuscript Number: PONE-D-26-14655

Title: “Exploring Sex-Specific Symptom Trajectories in Psychosis Following Metacognitive Training (MCT): evidence from a large-scale harmonized database”.

I appreciate the opportunity to review this compelling manuscript.

The present manuscript analyzed harmonized retrospective data from the international PERMEPSY consortium (573 participants with schizophrenia-spectrum disorders). Participants received Metacognitive Training (MCT) in either a clinical or a research setting. Psychotic symptoms were assessed using the PANSS. Repeated-measures ANOVAs were conducted to examine the effects of time, sex, and their interaction.

The results indicated that MCT was associated with significant reductions in positive, negative, general, and total symptoms. However, biological sex did not moderate changes in symptoms over time, indicating comparable improvement in men and women.

The results supported the effectiveness of MCT as a broadly applicable intervention, with comparable symptomatic improvement across genders. Although baseline differences were observed, biological sex did not influence the trajectory of change.

The authors highlighted the need to examine more nuanced sex/gender factors in future research.

The study has some strengths. It involved a large sample (N = 573), multicenter harmonized data, post hoc power analysis, and addresses a relevant issue.

The text is clear and well structured, although the title and abstract could explain the retrospective design and the absence of a control group.

The methodology is adequately described, and conclusions follow the logic of the main text.

Suggestions:

1. The authors may consider including more detailed baseline equivalence analyses, and discussing possible confounding variables (medication, duration of illness, therapeutic context). Moreover, authors should avoid language that suggests causality given the observational design.

2. My main concern regarding this manuscript is the lack of a convincing description of the actual contribution it makes to recent publications (some of which are from the same research group) that are not cited within the text. Although the paper aimed at exploring sex-specific symptom trajectories after MCT, the authors should consider discussing their results in relation to the following articles:

Gelner, H., et al. (2025). The role of demographic variables in the effectiveness of metacognitive training for psychosis: A meta-analysis. Schizophrenia Research, 283, 85-97. https://doi.org/10.1016/j.schres.2025.06.017

“MCT significantly reduced positive symptoms, delusions and improved cognitive insight. Women benefit more in cognitive insight in self-reflectiveness scale. However, no statistically significant gender differences were observed in other domains”.

Lamarca, M. et al. (2025). “The Role of Sex, Quality of Life, Self-Esteem and Cognitive Insight in Reducing Personal Stigma in Psychosis.” Clinical Psychology & Psychotherapy32, no. 6: e70171. https://doi.org/10.1002/cpp.70171

“Exploratory analyses suggested potential sex differences in stigma trajectories, with males showing greater improvements in internalized stigma”.

Verdaguer-Rodríguez, M., et al. (2026). Sex differences in neurocognitive response to metacognitive training in first-episode psychosis: Implications for personalized interventions. Arch. Womens. Ment. Health 29, 36. https://doi.org/10.1007/s00737-025-01637-3

“MCT enhances specific neurocognitive functions in FEP and shows promising effects for women in verbal memory processes. These findings underscore the importance of incorporating sex and diagnostic factors when tailoring early interventions for psychosis and highlight the potential of MCT as a personalized cognitive strategy”.

Goncalves, A. (2026). Examining sex as a potential moderator of metacognitive training for psychosis efficacy on cognitive insight and jumping to conclusions bias: Evidence from a large-scale harmonized database. Schizophrenia research. Cognition, 45, 100440. https://doi.org/10.1016/j.scog.2026.100440

“Contrary to previous FEP-specific findings, sex does not moderate MCT efficacy in heterogeneous clinical samples including established schizophrenia. These results may support MCT implementation without sex-specific adaptations in routine clinical practice”.

Minor issues:

Citation style across the manuscript is inconsistent, and should be adapted to the journal’s requirements.

Overall, the manuscript is well written, and its content is of interest.

Reviewer #3: Summary:

------------

This study presents a retrospective analysis of harmonized data from the PERMEPSY project, with an impressive sample size of N = 573, obtained from 22 different international studies which applied various versions of metacognitive training (MCT). A significant longitudinal reduction in symptoms across positive, negative, general, and total domains was observed. Biological sex was significantly associated with negative symptoms overall (lower levels among females), but no other symptoms. There was no significant interaction between sex and time (pre- vs. post-MCT), indicating no sex specific pre- vs. post-intervention effects.

The strengths of this study lie in the large sample size and the important topic it is investigating, especially in light of an emerging focus on sex-differences in the presentation and treatment of psychotic disorders. Furthermore, the article is very well and clearly written and structured, culminating in a nice and well-balanced discussion with suggestions for future research.

I have some concerns regarding the use of causal language, the analytical approach, and lacking information on the 22 studies included. These concerns could be addressed by providing some more information, reformulation of some of the statements included, and, potentially, re-analysis of the data.

Lacking information about the 22 studies:

-----------------------------------------------

The authors acknowledge that the different studies (in part) applied different variants on the MCT. More information on this should be provided, e.g. how many studies used a given method. Furthermore, the time interval between pre- and post-assessments is not mentioned anywhere in the manuscript. This should be provided, perhaps per study or method, depending on the extent to which time intervals differ or overlap.

I suggest adding a table with some descriptive data on the 22 samples including at minimum the following information: number of male/female participants, method of MCT used, duration of pre-post interval, country. Here, the authors could consider ‘merging’ overlapping information, e.g. tabulating by MCT method.

This information is important to accurately interpret the observed effects. It is possible that structural differences between the samples/centers (method/timing) confounded the sex variable (especially when there were systematic sex-differences across methods), perhaps overshadowing an actual sex*time interaction (see also next comment).

Analytical approach: rm ANOVA instead of linear-mixed effects models

-------------------------------------------------------------------------------

I was surprised to find the authors chose repeated measures ANOVA to investigate their hypotheses in the given sample. There appears to be missing data in the dataset (e.g., participants who only have pre-treatment scores). These cases would be discarded via listwise deletion in traditional ANOVAs, reducing statistical power and potentially introducing bias. Instead, linear-mixed effects analysis should be used, which allows participants with missing time points to be retained in the analysis, providing more robust and unbiased estimates.

Another benefit of linear-mixed effects analysis is that center/sample could be included as a random effect or MCT version/method as a fixed control variable (see also comment above). Currently, ‘time’ is treated as a discrete factor, but if the time interval differed between methods and/or centers, this introduces a confound. Controlling for center/method could partly take care of that. Same goes for differences in sex-distribution between centers/samples or MCT methods. Alternatively, the authors may consider providing sensitivity analyses per method, to demonstrate whether their results remain robust when isolating specific MCT variants or standardizing for follow-up durations.

A final benefit of linear-mixed effects analysis in my view is the obtainment of regression coefficients (and corresponding confidence intervals) which are interpretable in a more straight forward manner than eta^2, clearly indicating direction of effects and size in relation to unit-change. However, this may be a matter of taste and tradition, so not crucially important.

Causal language:

-------------------

Given the absence of a control group, the authors need to be careful when interpreting their time-effects as evidence for the effectiveness of MCT. Of course, in light of previous studies and general knowledge about the efficacy of MCT, I agree that the MCT intervention likely played a large role in symptom reduction over time.

However, strictly speaking this was not tested directly, and other factors between pre- and post-assessments may have contributed to symptom reduction over this time interval, such as medication adjustment, concurrent therapies, or regression to the mean (e.g., if some of the included studies used particularly high PANSS levels as an inclusion criterion at baseline).

In some places of the manuscript, the authors solve this elegantly, by using more accurate formulations such as ‘reductions in symptoms (…) following MCT’. However, in other sections of the manuscript, terms like ‘confirms’, ‘effective’, ‘effectiveness’/efficacy, or ‘underscores the robustness of MCT’ are used.

I suggest a thorough revision of the entire manuscript to ensure no causal claims are made, using a more careful terminology.

This limitation should also be acknowledged in the limitations section and/or be integrated into the discussion directly, for example here:

‘The significant main effect of Time across all PANSS subscales is consistent with previous literature demonstrating the efficacy of MCT in reducing psychotic symptoms severity [16,17]. These results are consistent with prior studies showing that MCT can effectively improve cognitive biases and symptom severity without directly confronting delusional content [16,22].’ --> Here, one could squeeze in a sentence to acknowledge the impossibility to claim causation, e.g. something along the lines of ‘while it cannot be ruled out that other factors accounted for some of the symptom reduction between pre- and post-treatment, findings are in in line with…’

Some more minor notes, per section:

------------------------------------------

Introduction:

--------------

‘treatment response [2]’ – Consider providing a better fitting/additional references to substantiate especially the latter point regarding treatment response.

Methods:

----------

For sociodemographic measures it is specified that they were recorded pre-treatment. Please also specify for PANSS: I assume just one assessment was conducted pre- and just one post-treatment? See also comment above on how additional information regarding the time interval between pre- and post-assessment should be provided.

Results:

---------

Please provide effect sizes for all tested effects, including non-significant ones (e.g., the Time × Sex interactions).

Post-hoc power analysis:

Post-hoc power analyses have been widely criticized, and do not provide valuable additional information. I understand why the authors wanted to provide them here, and acknowledge the challenge regarding power analyses when using retrospective data from multiple studies. However, I would suggest removing the post-hoc power analysis. Instead, the authors should provide confidence intervals around the observed effects. These can guide interpretation of both the observed significant and non-significant effects, thus potentially strengthening the authors’ argumentation. For example, if confidence intervals around the non-significant time*sex interaction are narrow and closely bound around zero (with upper bounds excluding even small-to-moderate effects) the authors could argue that any potential sex-specific effects are, if at all, so small that they don’t seem clinically meaningful.

Discussion:

------------

Formulation of the following sentence: ‘In addition, trends toward significance observed for PANSS_T and PANSS_P scores further suggest that females may experience slightly greater symptom reduction, but these effects did not reach statistical significance.’

--> If I understood correctly, this statement concerns the main effect. But the way it is formulated now implies an interaction effect (‘females may experience greater symptom reduction’). It should therefore be reformulated.

‘Small effect sizes observed across all PANSS domains warrant cautious interpretation’

--> please specify which effects are meant here, I assume of ‘time’?

**********

what does this mean?). If published, this will include your full peer review and any attached files.

If you choose “no”, your identity will remain anonymous but your review may still be made public.

**Do you want your identity to be public for this peer review?**  For information about this choice, including consent withdrawal, please see our Privacy Policy

Reviewer #1: No

Reviewer #2: No

Reviewer #3: No

**********

[NOTE: If reviewer comments were submitted as an attachment file, they will be attached to this email and accessible via the submission site. Please log into your account, locate the manuscript record, and check for the action link "View Attachments". If this link does not appear, there are no attachment files.]

To ensure your figures meet our technical requirements, please review our figure guidelines: https://journals.plos.org/plosone/s/figures

You may also use PLOS’s free figure tool, NAAS, to help you prepare publication quality figures: https://journals.plos.org/plosone/s/figures#loc-tools-for-figure-preparation.

NAAS will assess whether your figures meet our technical requirements by comparing each figure against our figure specifications.

# Author comment: Author response to Decision Letter 1

**DOI:** 10.1371/journal.pone.0356632.r002

18 Jun 2026

We have attached a formatted response to reviewers as a cover letter to improve the readibility of our changes, but please also find a copy of the applied changes below:

RESPONSE TO REVIEWERS

Manuscript: "Exploring Sex-Specific Symptom Trajectories in Psychosis Following Metacognitive Training (MCT): Evidence from a large-scale harmonized database"

Dear Editors and Reviewers,

We thank our reviewers for their detailed and constructive feedback, which has helped us strengthen the conceptual rationale and methodological transparency of the study, refine our interpretations, and improve the overall quality of the manuscript. We have provided detailed answers to all the issues raised in the revision letter below:

Journal and Editor Comments

1.Please ensure that your manuscript meets PLOS ONE's style requirements, including those for file naming. The PLOS ONE style templates can be found at

https://journals.plos.org/plosone/s/file?id=wjVg/PLOSOne_formatting_sample_main_body.pdf and

https://journals.plos.org/plosone/s/file?id=ba62/PLOSOne_formatting_sample_title_authors_affiliations.pdf

� We thank the journal and editor for this comment, we have fully reformatted the entire manuscript file and additional material to match the formats given in the above links. We hope the manuscript now meets the style requirements for this journal.

2. Thank you for stating the following in the Competing Interests section:

“I have read the journal's policy and the authors of this manuscript have the following competing interests: Steffen Moritz, one of the co-authors, is the main developer of Metacognitive Training (MCT) for psychosis. To minimize potential bias, he was not involved in data extraction, data harmonization, or statistical analyses for this study. No other authors have competing interests.”

Please confirm that this does not alter your adherence to all PLOS ONE policies on sharing data and materials, by including the following statement: "This does not alter our adherence to PLOS ONE policies on sharing data and materials.” (as detailed online in our guide for authors http://journals.plos.org/plosone/s/competing-interests). If there are restrictions on sharing of data and/or materials, please state these. Please note that we cannot proceed with consideration of your article until this information has been declared.

Please include your updated Competing Interests statement in your cover letter; we will change the online submission form on your behalf.

� We thank the journal and editor for this comment, we have updated the Conflicts of Interest section to reflect these issues:

p.24: This study is part of the PERMEPSY project, which involves Steffen Moritz, the main developer of MCT for psychosis. To mitigate potential conflicts of interest, Steffen Moritz was not involved in data extraction, data harmonization, or statistical analyses for this study. This does not alter our adherence to PLOS ONE policies on sharing data and materials. However, due to the retrospective nature of this study, and the data collection heterogeneity in terms of informed consent received by various studies, the consortium has been advised not to share the retrospective database in open access.

3. Please note that funding information should not appear in any section or other areas of your manuscript. We will only publish funding information present in the Funding Statement section of the online submission form. Please remove any funding-related text from the manuscript.

� We thank the journal and editor for this comment, we have removed the following section from page 23:

Fundings

The PERMEPSY project was supported under the frame of ERA PerMed (ANR-22-PERM-0009-05) by: Instituto de Salud Carlos III (ISCIII), Spain; German Federal Ministry of Education and Research (BMBF), Germany; Agence Nationale de la Recherche (ANR), France; National Centre for Research and Development (NCBR), Poland; Agencia Nacional de Investigación y Desarrollo (ANID), Chile. The funding agency is not involved in the execution of this study or in the evaluation of the results.

� We would like to update this section to reflect the following:

This work is part of the European ERAPERMED 2022-292 call for international collaboration projects of the 2021–2023 Strategic Action in Health funded by European Union - Next Generation EU resources under the Recovery, Transformation and Resilience Plan, supported as grant AC22/0010 by the Instituto de Salud Carlos III (ISCIII) in Spain. The PERMEPSY project was supported under the frame of ERA PerMed by: Instituto de Salud Carlos III (ISCIII), Spain, Grant numbers AC22/00010 & AC22/0053; German Federal Ministry of Education and Research (BMBF), Germany, Grant number 01KU2306; Agence Nationale de la Recherche (ANR), France, Grant number ANR-22-PERM-0009-05.; National Centre for Research and Development (NCBR) Grant number: PerMed/V/82/PERMEPSY/2023, Poland; Agencia Nacional de Investigación y Desarrollo (ANID), Chile, Grant number REC+ERAPERMED2022-292.

4. We note that you have indicated that there are restrictions to data sharing for this study. PLOS only allows data to be available upon request if there are legal or ethical restrictions on sharing data publicly. For more information on unacceptable data access restrictions, please see http://journals.plos.org/plosone/s/data-availability#loc-unacceptable-data-access-restrictions.

Before we proceed with your manuscript, please address the following prompts:

a) If there are ethical or legal restrictions on sharing a de-identified data set, please explain them in detail (e.g., data contain potentially identifying or sensitive patient information, data are owned by a third-party organization, etc.) and who has imposed them (e.g., a Research Ethics Committee or Institutional Review Board, etc.). Please also provide contact information for a data access committee, ethics committee, or other institutional body to which data requests may be sent.

b) If there are no restrictions, please upload the minimal anonymized data set necessary to replicate your study findings to a stable, public repository and provide us with the relevant URLs, DOIs, or accession numbers. For a list of recommended repositories, please see

https://journals.plos.org/plosone/s/recommended-repositories. You also have the option of uploading the data as Supporting Information files, but we would recommend depositing data directly to a data repository if possible.

We will update your Data Availability statement on your behalf to reflect the information you provide.

� We thank the journal and editor for this comment, we have updated the Data Availability Statement section to reflect our data-sharing restrictions. The data was collected across 22 international databases with different ethical constraints, and when we requested data from third party researchers (i.e., outside our consortium), they agreed to share it in anonymized format given that we will not share the data openly once harmonized. We were advised by Data Protection Officers across our participating centres not to share the harmonized dataset since we do not have complete information regarding the collection of informed consent in all centres, only those we collected ourselves (8 databases out of 22). We are aware that all data was collected with appropriate ethics regulation and informed consent, but we are unsure if international data external to PERMEPSY members was collected from patients who gave their consent to data being publicly available. To be safe, we decided as a consortium not to share the full retrospective database in open access. This is summarized now in the Data Availability Statement:

p.24: The PERMEPSY harmonized dataset underlying this study is not publicly available due to ethical and data governance constraints across the contributing international studies. Researchers wishing to access the dataset may submit a formal request to the PERMEPSY consortium (www.permepsy.org), where requests are reviewed by the steering committee in accordance with applicable ethical guidelines. The data harmonization pipeline and variable structure are fully documented in König et al. (2025a) and the associated Python scripts are publicly available at https://gitlab-rdlab.cs.upc.edu/soco-permepsy/harmonization. The complete Jamovi analysis template (without patient data) has been deposited on the Open Science Framework: https://osf.io/a8yf7/overview?view_only=a1a1ac93340543058ca396ee75aa3b4e

5. In the online submission form you indicate that your data is not available for proprietary reasons and have provided a contact point for accessing this data. Please note that your current contact point is a co-author on this manuscript. According to our Data Policy, the contact point must not be an author on the manuscript and must be an institutional contact, ideally not an individual. Please revise your data statement to a non-author institutional point of contact, such as a data access or ethics committee, and send this to us via return email. Please also include contact information for the third party organization, and please include the full citation of where the data can be found.

� We thank the journal and editor for this comment, we have updated the Data Availability Statement section to reflect our data-sharing restrictions, and added the contact point for the consortium data management committee. Additionally, since this project is coordinated by the Parc Sanitari Sant Joan de Déu, their Data Management Officer can be contacted at: claudia.garrido@sjd.es or their Data Protection Officer at: oficina_dpd@pssjd.org). The remaning information requested is updated in the Data Availability Statement:

p.24: The PERMEPSY harmonized dataset underlying this study is not publicly available due to ethical and data governance constraints across the contributing international studies. Researchers wishing to access the dataset may submit a formal request to the PERMEPSY consortium (www.permepsy.org), where requests are reviewed by the steering committee in accordance with applicable ethical guidelines. The data harmonization pipeline and variable structure are fully documented in König et al. (2025a) and the associated Python scripts are publicly available at https://gitlab-rdlab.cs.upc.edu/soco-permepsy/harmonization. The complete Jamovi analysis template (without patient data) has been deposited on the Open Science Framework: https://osf.io/a8yf7/overview?view_only=a1a1ac93340543058ca396ee75aa3b4e

6. If the reviewer comments include a recommendation to cite specific previously published works, please review and evaluate these publications to determine whether they are relevant and should be cited. There is no requirement to cite these works unless the editor has indicated otherwise.

� We thank you for this comment, and have taken it into account when revising the manuscript with the reviewer comments.

Additional Editor Comments:

The revisions mainly concern the analytical approach, interpretation of findings, and handling of heterogeneity across studies. In particular, the reviewers requested either a re-analysis using linear mixed-effects models or a stronger justification for the current analytical strategy, as well as a more cautious interpretation of symptom changes given the absence of a control group. Additional recommended revisions include expanding methodological details regarding the included studies and MCT variants, clarifying the handling of missing data, reporting confidence intervals and effect sizes more systematically, and integrating recent related literature into the discussion.

� We thank the Editor for summarizing the concerns from the reviewers, we agree with the requested revisions and below have outlined all changes we have made to the revised manuscript. Thank you for improving our work.

Reviewer #1

This manuscript examines whether biological sex moderates symptom trajectories following Metacognitive Training (MCT) in individuals with schizophrenia spectrum disorders, using a large harmonized retrospective dataset (n = 573) derived from the PERMEPSY consortium.

The study addresses a relevant and timely question, given increasing interest in sex differences in psychosis and the dissemination of scalable psychological interventions. The use of a large international dataset and standardized outcome measures (PANSS) represents a clear strength. The manuscript is generally well structured and written in clear scientific English.

However, several important issues limit the strength of the conclusions. These relate primarily to design limitations, analytic strategy, handling of heterogeneity, and interpretation of findings. While the study meets several PLOS One criteria (originality, clarity), further clarification and methodological strengthening are needed to ensure that conclusions are fully supported by the data.

� We thank the reviewer for their careful evaluation of our manuscript and for highlighting both its strengths and areas for improvement. We appreciate the recognition of the relevance of examining sex differences in response to Metacognitive Training (MCT), as well as the strengths of the large, international PERMEPSY dataset and the use of standardized PANSS outcomes. We agree with the reviewer that several important aspects regarding design limitations, analytic strategy, heterogeneity, and interpretation require further clarification and strengthening to ensure that our conclusions are fully supported by the data. In response, we have revised the manuscript accordingly and provide a detailed, point-by-point response below:

#1. Lack of Control Condition and Causal Interpretation. The most important limitation concerns the absence of a control group. The manuscript repeatedly refers to MCT as 'effective' in reducing symptoms. However, the design is retrospective and uncontrolled, meaning that symptom reduction cannot be attributed specifically to MCT. This is particularly relevant given naturalistic symptom fluctuation, regression to the mean, and concurrent treatments (e.g., medication, other psychosocial interventions). The authors should substantially tone down causal language and reframe findings as 'symptom changes observed following MCT exposure' rather than evidence of effectiveness."

� We thank the reviewer for this important methodological remark. We fully agree that the absence of a control group precludes causal attribution, and we have conducted a thorough revision of the entire manuscript to eliminate causal language. Terms such as "effective", "effectiveness", "confirms" and "underscores the robustness" have been replaced with more appropriate formulations such as "consistent with prior evidence", "associated with reductions", and "symptom changes observed following MCT". This revision applies to the abstract, results, discussion, and conclusions sections.

#2. Heterogeneity of the Dataset (Major Threat to Internal Validity). The PERMEPSY dataset combines 22 studies, multiple countries, different illness stages, and multiple MCT formats (group, MCT+, MCT-N). This introduces substantial clinical and methodological heterogeneity, which is not adequately controlled. Key concerns:

- No modelling of study-level clustering effects

- No adjustment for MCT format differences

- No control for illness stage, duration, or baseline severity

- No multilevel modelling despite nested data structure

At minimum, the authors should acknowledge this as a major limitation more explicitly, and consider including study as a random factor (if possible), or sensitivity analyses (e.g., restricting to subsamples).

� We sincerely thank the reviewer for this methodologically important concern, which we fully acknowledge. We agree that linear mixed-effects models with study as a random effect would theoretically represent a more appropriate framework for nested data of this structure. However, implementing such models is structurally precluded by the nature of the PERMEPSY harmonized database itself. As described in König et al. (2025a), the pipeline did not retain participant identifiers or study-of-origin labels, as the primary objective was to integrate pre- and post-treatment evaluat

# Peer review: Decision Letter 1

**DOI:** 10.1371/journal.pone.0356632.r003

**Authors:** César González-Blanch

14 Jul 2026

Dear Dr. Lamarca,

Thank you for submitting your manuscript to PLOS ONE. After careful consideration, we feel that it has merit but does not fully meet PLOS ONE’s publication criteria as it currently stands. Therefore, we invite you to submit a revised version of the manuscript that addresses the points raised during the review process.

The manuscript has improved substantially, and the authors have addressed most of the concerns raised in the previous round of review. The revised version is clearer, more transparent regarding its methodological limitations, and more cautious in its interpretation of the findings. Nevertheless, the remaining issues identified by Reviewers 1 and 3 should be addressed before the manuscript is accepted. These issues appear addressable without major changes to the study design or additional analyses.

Please submit your revised manuscript by Aug 28 2026 11:59PM. If you will need more time than this to complete your revisions, please reply to this message or contact the journal office at plosone@plos.org. When you're ready to submit your revision, log on to https://www.editorialmanager.com/pone/ and select the 'Submissions Needing Revision' folder to locate your manuscript file.

- A letter that responds to each point raised by the academic editor and reviewer(s). You should upload this letter as a separate file labeled 'Response to Reviewers'.
- A marked-up copy of your manuscript that highlights changes made to the original version. You should upload this as a separate file labeled 'Revised Manuscript with Track Changes'.
- An unmarked version of your revised paper without tracked changes. You should upload this as a separate file labeled 'Manuscript'.

If you would like to make changes to your financial disclosure, please include your updated statement in your cover letter. Guidelines for resubmitting your figure files are available below the reviewer comments at the end of this letter.

If applicable, we recommend that you deposit your laboratory protocols in protocols.io to enhance the reproducibility of your results. Protocols.io assigns your protocol its own identifier (DOI) so that it can be cited independently in the future. For instructions see: https://journals.plos.org/plosone/s/submission-guidelines#loc-laboratory-protocols. Additionally, PLOS ONE offers an option for publishing peer-reviewed Lab Protocol articles, which describe protocols hosted on protocols.io. Read more information on sharing protocols at https://plos.org/protocols?utm_medium=editorial-email&utm_source=authorletters&utm_campaign=protocols.

As the corresponding author, your ORCID iD is verified in the submission system and will appear in the published article. PLOS supports the use of ORCID, and we encourage all coauthors to register for an ORCID iD and use it as well. Please encourage your coauthors to verify their ORCID iD within the submission system before final acceptance, as unverified ORCID iDs will not appear in the published article. *Only*  the individual author can complete the verification step; PLOS staff *cannot*  verify ORCID iDs on behalf of authors.

We look forward to receiving your revised manuscript.

Kind regards,

César González-Blanch, PhD

Academic Editor

PLOS One

Journal Requirements:

1. If the reviewer comments include a recommendation to cite specific previously published works, please review and evaluate these publications to determine whether they are relevant and should be cited. There is no requirement to cite these works unless the editor has indicated otherwise.

2. Please review your reference list to ensure that it is complete and correct. If you have cited papers that have been retracted, please include the rationale for doing so in the manuscript text, or remove these references and replace them with relevant current references. Any changes to the reference list should be mentioned in the rebuttal letter that accompanies your revised manuscript. If you need to cite a retracted article, indicate the article’s retracted status in the References list and also include a citation and full reference for the retraction notice.

[Note: HTML markup is below. Please do not edit.]

Reviewers' comments:

Reviewer's Responses to Questions

**Comments to the Author**

Reviewer #1: (No Response)

Reviewer #2: All comments have been addressed

Reviewer #3: (No Response)

**********

2. Is the manuscript technically sound, and do the data support the conclusions?

Reviewer #1: Yes

Reviewer #2: Yes

Reviewer #3: Yes

**********

3. Has the statistical analysis been performed appropriately and rigorously?

Reviewer #1: Yes

Reviewer #2: Yes

Reviewer #3: Yes

**********

4. Have the authors made all data underlying the findings in their manuscript fully available?

The PLOS Data policy

Reviewer #1: Yes

Reviewer #2: Yes

Reviewer #3: No

**********

5. Is the manuscript presented in an intelligible fashion and written in standard English?

Reviewer #1: Yes

Reviewer #2: Yes

Reviewer #3: Yes

**********

Reviewer #1: Thank you for the careful and thorough revision of the manuscript. The authors have addressed the majority of the concerns raised in the previous review round. In particular, I appreciate the efforts to:

1. Remove or substantially reduce causal language throughout the manuscript.

2. Clarify the retrospective and uncontrolled nature of the study.

3. Expand discussion of dataset heterogeneity.

4. Provide additional information regarding the contributing studies.

5. Conduct and report a sensitivity analysis restricted to first-episode psychosis participants.

6. Clarify the distinction between biological sex and gender.

7. Improve transparency regarding data availability and governance restrictions.

Overall, the manuscript is now substantially stronger, more transparent, and better aligned with the conclusions supported by the data.

I have only a few remaining comments.

1. The authors explain that study-of-origin identifiers and MCT format information were not retained during harmonization, making multilevel modelling impossible. I appreciate this clarification. However, this is not merely a statistical limitation but also a limitation of the harmonization strategy itself. The current wording may leave readers with the impression that mixed-effects modelling was impossible due to the dataset structure, whereas in fact the harmonization process eliminated variables that would have allowed modelling study-level clustering and intervention-format effects. I would recommend slightly strengthening the wording in the limitations section to explicitly acknowledge that future harmonization initiatives should preserve study-level identifiers and intervention-format information to permit more rigorous secondary analyses.

2. The newly added baseline analyses indicate significant differences between males and females in age, diagnostic distribution, positive symptoms, and negative symptoms. These differences are important because they complicate interpretation of subsequent symptom trajectories. Although the discussion now mentions these findings, I would encourage the authors to elaborate slightly further on whether such baseline imbalances could potentially mask or dilute sex-by-time interactions. I am not requesting additional analyses, merely a somewhat more explicit acknowledgement of this interpretive limitation.

3. I appreciate the additional discussion regarding statistical versus clinical significance. However, the manuscript still relies almost exclusively on group-level statistical effects. Because responder analyses and clinically meaningful change indices could not be calculated, I suggest adding one sentence explicitly stating that the clinical importance of the observed symptom reductions cannot be determined from the current dataset. This would further align interpretation with the available evidence.

4. The revised data availability section is substantially improved and provides a clearer justification for restricted access. I appreciate the authors’ efforts to provide access procedures, analysis scripts, harmonization pipeline documentation, and OSF materials. I have no further concerns regarding transparency from a scientific perspective, although final evaluation of compliance with PLOS ONE data-sharing policies should remain an editorial decision.

Reviewer #2: In my opinion, the authors have made an important effort to improve the original submission, resulting in a ready-to-publish manuscript.

Reviewer #3: Overall, the manuscript has been substantially improved. The causal language has been toned down, the analytical approach is explained and justified more clearly, the corresponding limitations have been addressed, and a helpful overview over the different included studies has been provided.

However, there is one section in the discussion that appears a bit unclear and somewhat self-contradictory. This is also related to the fact that the authors seem to have misunderstood my previous comments about confidence intervals for effect sizes. For clarification:

In the previous review, I suggested adding confidence intervals to the obtained effect sizes, i.e. main and interaction effects), as this would provide more meaningful information than a post-hoc power analysis. I appreciate the authors’ responsiveness to this comment, and the newly added table of Estimated Marginal Means and 95% confidence intervals is an illustrative and informative addition to the manuscript. However, providing confidence intervals simply for the individual cells (time x sex for each PANSS subscale) does not address the original issue. The here provided cell-level confidence intervals indicate the precision of group scores at a single time point, but do not reflect the precision or uncertainty of the actual main effects and interaction effects derived from the repeated-measures ANOVA models. Instead, the authors may want to look into ways to calculate confidence intervals for the reported partial eta-squared estimates. (see, e.g., for an R-based approach: Guide to Effect Sizes and Confidence Intervals, Jané et al., 2024, https://osf.io/d8c4g/; though other approaches are possible, of course).

.... Side note: I also noticed that the degrees of freedom are missing for the reported F-statistics throughout the manuscript. These should be added, as it is currently unclear exactly how many participants were included in each respective analysis (given different proportions of missing data pre- and post-treatment).

Furthermore, the discussion paragraph where the confidence intervals are mentioned is currently written in a somewhat confusing and contradictory manner, making it hard to follow: from p. 19, line 403 (‘Interestingly, while no significant sex ….’) to p. 20, line 418/419 (‘…did not reach statistical significance.’). Here, the authors alternate between stating significant differences, then stating that overlapping CIs show there are no differences, and then pointing to non-significant 'trends' to suggest there might be a difference. This section needs to be streamlined.

**********

what does this mean?). If published, this will include your full peer review and any attached files.

If you choose “no”, your identity will remain anonymous but your review may still be made public.

**Do you want your identity to be public for this peer review?**  For information about this choice, including consent withdrawal, please see our Privacy Policy

Reviewer #1: No

Reviewer #2: **Yes:**  Josep Pena-Garijo

Reviewer #3: No

**********

[NOTE: If reviewer comments were submitted as an attachment file, they will be attached to this email and accessible via the submission site. Please log into your account, locate the manuscript record, and check for the action link "View Attachments". If this link does not appear, there are no attachment files.]

To ensure your figures meet our technical requirements, please review our figure guidelines: https://journals.plos.org/plosone/s/figures

You may also use PLOS’s free figure tool, NAAS, to help you prepare publication quality figures: https://journals.plos.org/plosone/s/figures#loc-tools-for-figure-preparation.

NAAS will assess whether your figures meet our technical requirements by comparing each figure against our figure specifications.

# Author comment: Author response to Decision Letter 2

**DOI:** 10.1371/journal.pone.0356632.r004

30 Jul 2026

RESPONSE TO REVIEWERS

Manuscript: "Exploring Sex-Specific Symptom Trajectories in Psychosis Following Metacognitive Training (MCT): Evidence from a large-scale harmonized database"

Dear Editors and Reviewers,

We thank our reviewers for their detailed and constructive feedback, which has helped us strengthen the conceptual rationale and methodological transparency of the study, refine our interpretations, and improve the overall quality of the manuscript. We have provided detailed answers to all the issues raised in the revision letter below:

Journal and Editor Comments

1. If the reviewer comments include a recommendation to cite specific previously published works, please review and evaluate these publications to determine whether they are relevant and should be cited. There is no requirement to cite these works unless the editor has indicated otherwise.

� We thank you for this comment and have taken it into account when revising the manuscript with the reviewer comments.

2. Please review your reference list to ensure that it is complete and correct. If you have cited papers that have been retracted, please include the rationale for doing so in the manuscript text, or remove these references and replace them with relevant current references. Any changes to the reference list should be mentioned in the rebuttal letter that accompanies your revised manuscript. If you need to cite a retracted article, indicate the article’s retracted status in the References list and also include a citation and full reference for the retraction notice.

� We thank the editor for this reminder. We have carefully reviewed our reference list and verified all DOIs using the Retraction Watch Database (https://retractiondatabase.org/RetractionSearch.aspx); no retracted articles were identified among our citations. During this verification, we identified three duplicate entries, which have been corrected: references 12 and 17 (Salas-Sender et al., 2020, now reference 12), references 16 and 22 (Goncalves et al., 2025, now reference 16), and references 19 and 28 (König et al., 2025, now reference 18). The reference list has been updated accordingly.

Reviewer #1

Thank you for the careful and thorough revision of the manuscript. The authors have addressed the majority of the concerns raised in the previous review round. In particular, I appreciate the efforts to:

1. Remove or substantially reduce causal language throughout the manuscript.

2. Clarify the retrospective and uncontrolled nature of the study.

3. Expand discussion of dataset heterogeneity.

4. Provide additional information regarding the contributing studies.

5. Conduct and report a sensitivity analysis restricted to first-episode psychosis participants.

6. Clarify the distinction between biological sex and gender.

7. Improve transparency regarding data availability and governance restrictions.

Overall, the manuscript is now substantially stronger, more transparent, and better aligned with the conclusions supported by the data.

I have only a few remaining comments.

� We sincerely thank the reviewer for the careful evaluation of our revised manuscript and for recognizing the extensive efforts made to address the concerns raised during the previous review round. We greatly appreciate the positive assessment that the manuscript is now stronger, more transparent, and better aligned with the conclusions supported by the data. We are grateful for the reviewer’s constructive feedback throughout the review process, which has significantly contributed to improving the scientific rigor, clarity, and transparency of this work. We have carefully considered the remaining comments and have addressed each of them in detail below.

1. The authors explain that study-of-origin identifiers and MCT format information were not retained during harmonization, making multilevel modelling impossible. I appreciate this clarification. However, this is not merely a statistical limitation but also a limitation of the harmonization strategy itself. The current wording may leave readers with the impression that mixed-effects modelling was impossible due to the dataset structure, whereas in fact the harmonization process eliminated variables that would have allowed modelling study-level clustering and intervention-format effects. I would recommend slightly strengthening the wording in the limitations section to explicitly acknowledge that future harmonization initiatives should preserve study-level identifiers and intervention-format information to permit more rigorous secondary analyses.

� We thank the reviewer for this valuable observation. We agree that the inability to model study-level clustering and intervention-format effects reflects not only a statistical limitation of the final dataset but also a limitation of the harmonization strategy adopted during database construction. We have revised the limitations section to clarify that study-of-origin identifiers and MCT format information were not retained during harmonization, which prevented the application of multilevel modelling and stratified analyses by intervention format. We further acknowledge that future harmonization initiatives should preserve these variables to facilitate more rigorous secondary analyses, including the modelling of study-level clustering and intervention-specific effects.

Specifically, we have added the following sentence in page 19: “This limitation reflects not only constraints of the final analytic dataset but also a limitation of the harmonization strategy itself. Future harmonization initiatives should preserve study-level identifiers and intervention-format information to enable more rigorous secondary analyses and better account for sources of between-study heterogeneity.”

2. The newly added baseline analyses indicate significant differences between males and females in age, diagnostic distribution, positive symptoms, and negative symptoms. These differences are important because they complicate interpretation of subsequent symptom trajectories. Although the discussion now mentions these findings, I would encourage the authors to elaborate slightly further on whether such baseline imbalances could potentially mask or dilute sex-by-time interactions. I am not requesting additional analyses, merely a somewhat more explicit acknowledgement of this interpretive limitation.

� We thank the reviewer for this insightful comment. We have expanded the Limitations Discussion section to explicitly recognize that baseline imbalances between males and females may have reduced our ability to detect sex-by-time interactions. In particular, differences in initial symptom severity and diagnostic composition may have introduced additional variability in change scores, potentially masking or diluting subtle sex-specific treatment effects. We have added this consideration as an important interpretive limitation of the study.

Page 20: “Fifth, the observed baseline imbalances in age, diagnostic distribution, and symptom severity between males and females may have complicated the interpretation of longitudinal symptom trajectories. Differences in initial clinical characteristics could have increased variability in treatment-related change, potentially masking or diluting subtle Time × Sex interactions. Consequently, the absence of significant sex moderation effects should not be interpreted as definitive evidence that sex plays no role in treatment response, but rather as an indication that any such effects were not detectable within the context of the heterogeneous baseline profiles represented in the present sample.”

3. I appreciate the additional discussion regarding statistical versus clinical significance. However, the manuscript still relies almost exclusively on group-level statistical effects. Because responder analyses and clinically meaningful change indices could not be calculated, I suggest adding one sentence explicitly stating that the clinical importance of the observed symptom reductions cannot be determined from the current dataset. This would further align interpretation with the available evidence.

� We thank the reviewer for this valuable suggestion. We have added a sentence to explicitly acknowledge this limitation and to ensure that our interpretation remains closely aligned with the evidence available from the current dataset.

This sentence can be found in page 18: “Furthermore, although statistically significant symptom reductions were observed, the absence of responder analyses and clinically meaningful change indicators prevents conclusions regarding the clinical importance of these changes at the individual patient level.”

4. The revised data availability section is substantially improved and provides a clearer justification for restricted access. I appreciate the authors’ efforts to provide access procedures, analysis scripts, harmonization pipeline documentation, and OSF materials. I have no further concerns regarding transparency from a scientific perspective, although final evaluation of compliance with PLOS ONE data-sharing policies should remain an editorial decision.

� We thank the reviewer for this positive assessment of the revised Data Availability Statement. We agree that the final evaluation of compliance with PLOS ONE data-sharing policies falls within the remit of the editorial team, and we remain available to provide any additional information or clarification that may be required by the editors.

We sincerely thank the reviewer for the thoughtful, constructive, and rigorous feedback provided throughout the review process. We greatly appreciate the time and effort invested in evaluating our work and helping us improve the manuscript.

Reviewer #2

5. In my opinion, the authors have made an important effort to improve the original submission, resulting in a ready-to-publish manuscript.

� We sincerely thank the reviewer for the positive assessment of our manuscript. We greatly appreciate the recognition of the efforts made to address the reviewers’ comments and improve the quality of the original submission. We are pleased that the revisions have strengthened the manuscript and that, in the reviewer's opinion, it is now suitable for publication. We are grateful for the time and consideration dedicated to the evaluation of our work and for the constructive feedback provided throughout the review process.

Reviewer #3

Overall, the manuscript has been substantially improved. The causal language has been toned down, the analytical approach is explained and justified more clearly, the corresponding limitations have been addressed, and a helpful overview over the different included studies has been provided. manuscript.

� We sincerely thank the reviewer for their evaluation of our revised manuscript. We greatly appreciate the recognition that the revisions have improved the clarity, transparency, and overall rigor of the study. We are also grateful for the reviewer’s continued careful evaluation of the manuscript and for identifying the remaining areas that require clarification. We have carefully considered the comments and we address these points in detail below.

6. However, there is one section in the discussion that appears a bit unclear and somewhat self-contradictory. This is also related to the fact that the authors seem to have misunderstood my previous comments about confidence intervals for effect sizes. For clarification: In the previous review, I suggested adding confidence intervals to the obtained effect sizes, i.e. main and interaction effects), as this would provide more meaningful information than a post-hoc power analysis. I appreciate the authors’ responsiveness to this comment, and the newly added table of Estimated Marginal Means and 95% confidence intervals is an illustrative and informative addition to the manuscript. However, providing confidence intervals simply for the individual cells (time x sex for each PANSS subscale) does not address the original issue. The here provided cell-level confidence intervals indicate the precision of group scores at a single time point, but do not reflect the precision or uncertainty of the actual main effects and interaction effects derived from the repeated-measures ANOVA models. Instead, the authors may want to look into ways to calculate confidence intervals for the reported partial eta-squared estimates. (see, e.g., for an R-based approach: Guide to Effect Sizes and Confidence Intervals, Jané et al., 2024, https://osf.io/d8c4g/; though other approaches are possible, of course).

.... Side note: I also noticed that the degrees of freedom are missing for the reported F-statistics throughout the manuscript. These should be added, as it is currently unclear exactly how many participants were included in each respective analysis (given different proportions of missing data pre- and post-treatment).

� We thank the reviewer for this important clarification, and apologize for the misunderstanding regarding the nature of the requested confidence intervals in the previous revision round. We have now computed 90% confidence intervals for the partial eta-squared (η²p) values associated with each main and interaction effect from the repeated-measures ANOVA models, rather than for the estimated marginal means alone. These confidence intervals were computed using the non-central F-distribution method (Steiger, 2004), implemented in the effectsize R package (Ben-Shachar et al., 2020), following the approach recommended in the guide cited by the reviewer (Jané et al., 2024). As recommended in that same guide, we opted for 90%, rather than 95%, confidence intervals for η²p, given that F-tests are inherently one-sided and a 95% CI can therefore include zero even when the corresponding omnibus test is statistically significant. We have also added the degrees of freedom for all reported F-statistics throughout the Results section, addressing the reviewer's side note regarding the number of participants included in each analysis.

These changes are reflected in the Results section and in the Methods section (page 12): For each model, we report partial eta-squared (η²p) as the effect size measure for the main and interaction effects, together with 90% confidence intervals computed using the non-central F-distribution method, implemented in the effectsize R package. Ninety percent, rather than 95%, confidence intervals were used for η²p because F-tests are inherently one-sided, meaning that a 95% CI can include zero even when the corresponding omnibus test is statistically significant”

� Additionally, while cross-checking these values against the original jamovi output during this revision, we identified and corrected a transcription error in the reported F-statistic for the Time × Sex interaction on PANSS_T (previously reported as F = 0.474; corrected to F = 0.514).

Furthermore, the discussion paragraph where the confidence intervals are mentioned is currently written in a somewhat confusing and contradictory manner, making it hard to follow: from p. 19, line 403 (‘Interestingly, while no significant sex ….’) to p. 20, line 418/419 (‘…did not reach statistical significance.’). Here, the authors alternate between stating significant differences, then stating that overlapping CIs show there are no differences, and then pointing to non-significant 'trends' to suggest there might be a difference. This section needs to be streamlined

� We thank the reviewer for this comment and agree that this paragraph required streamlining. We have substantially revised it to remove the alternating and potentially contradictory reasoning identified by the reviewer. Specifically, we no longer rely on the overlap of confidence intervals around estimated marginal means (Supplementary Table S2) to infer the absence of a sex-specific effect, nor do we refer to non-significant "trends" to suggest a possible effect in the opposite direction. Instead, the revised paragraph reports the pattern of significance for the main effect of Sex and the Time × Sex interaction in a single, non-repetitive statement, and separately situates the magnitude of the Time effect relative to standardized effect sizes (Cohen's dz) reported in prior MCT meta-analyses, which we consider a mor

Attachment Submitted filename: Response To Reviewers 2.docx

# Editor report: Decision Letter 2

**DOI:** 10.1371/journal.pone.0356632.r005

**Authors:** César González-Blanch

5 Aug 2026

Exploring sex-specific symptom trajectories in psychosis following metacognitive training (MCT): evidence from a large-scale retrospective harmonized database.

PONE-D-26-14655R2

Dear Dr. Lamarca,

We’re pleased to inform you that your manuscript has been judged scientifically suitable for publication and will be formally accepted for publication once it meets all outstanding technical requirements.

Within one week, you’ll receive an e-mail detailing the required amendments. When these have been addressed, you’ll receive a formal acceptance letter and your manuscript will be scheduled for publication.

An invoice will be generated when your article is formally accepted. Please note, if your institution has a publishing partnership with PLOS and your article meets the relevant criteria, all or part of your publication costs will be covered. Please make sure your user information is up-to-date by logging into Editorial Manager at Editorial Manager® and clicking the ‘Update My Information' link at the top of the page. For questions related to billing, please contact billing support.

If your institution or institutions have a press office, please notify them about your upcoming paper to help maximize its impact. If they’ll be preparing press materials, please inform our press team as soon as possible -- no later than 48 hours after receiving the formal acceptance. Your manuscript will remain under strict press embargo until 2 pm Eastern Time on the date of publication. For more information, please contact onepress@plos.org.

Kind regards,

César González-Blanch, PhD

Academic Editor

PLOS One

Additional Editor Comments (optional):

Reviewers' comments:
